# Gabpα‐Pparγ Complex Determines Glycolytic Capacity and Lactic Acid Homeostasis in Brown Fat

**DOI:** 10.1002/advs.202517426

**Published:** 2025-11-23

**Authors:** Zhihan Wang, Huanyu Wang, Qianqian Kang, Ruping Pan, Rui He, Min Yang, Jiadai Liu, Xuemin Peng, Yuyu Xie, Hongyan Deng, Wenshe Wang, Zengzhe Zhu, Jing Ge, Yulian Liu, Ronghui Gao, Yaming Guo, Peng Yu, Limeng Pan, Danpei Li, Pema Maretich, Xiaoping Luo, Xuefeng Yu, Yong Chen

**Affiliations:** ^1^ Division of Endocrinology Department of Internal Medicine Tongji Hospital Tongji Medical College and State Key Laboratory for Diagnosis and Treatment of Severe Zoonotic Infectious Diseases Huazhong University of Science and Technology Wuhan 430030 China; ^2^ Laboratory of Endocrinology and Metabolism Ministry of Education Key Laboratory of Vascular Aging Tongji Hospital Tongji Medical College Huazhong University of Science and Technology Wuhan 430000 China; ^3^ Department of Pediatrics Hubei Provincial Key Laboratory of Pediatric Genetic Metabolic and Endocrine Rare Diseases Hubei Provincial Clinical Research Center for Children's Growth and Development and Metabolic Diseases Tongji Hospital Tongji Medical College Huazhong University of Science and Technology Wuhan 430030 China; ^4^ Department of Nuclear Medicine Tongji Hospital Tongji Medical College Huazhong University of Science and Technology Wuhan 430030 China; ^5^ Research Laboratory of Electronics and Department of Biology Massachusetts Institute of Technology Cambridge MA 02139 USA; ^6^ Hubei Clinical Medical Research Center for Endocrinology and Metabolic Diseases Branch of National Clinical Research Center for Metabolic Diseases Hubei 430000 China

**Keywords:** brown fat, glycolysis, lactate, transcriptional regulation

## Abstract

Glycolysis in brown adipose tissue (BAT) plays a critical role in fueling thermogenesis. However, the transcriptional control of glycolysis in brown fat remains poorly understood. Here, GA binding protein alpha chain (Gabpα) is identified as a key transcriptional regulator that sustains the glycolytic capacity of brown adipocytes. Gabpα is preferentially expressed in BAT, yet BAT‐specific ablation of Gabpα substantially impairs glycolytic flux and heat production, leading to reduced glucose tolerance and impaired cold tolerance. Mechanistically, the C‐terminus of the Gabpα protein directly interacts with peroxisome proliferator‐activated receptor‐γ (Pparγ) and synergistically promotes transcription of the glycolytic gene *enolase 1* (*Eno1*). Disruption of the Gabpα–Pparγ interaction in BAT significantly suppresses glycolysis, reduces energy expenditure, and induces cold intolerance in mice. Notably, inhibition of Gabpα‐Pparγ binding also decreases lactic acid concentration and downregulates lactate dehydrogenase (Ldh) expression, resulting in the suppression of uncoupling protein 1 (Ucp1) expression and thermogenesis. Conversely, adipose‐specific overexpression of Gabpα markedly enhances BAT glycolytic and thermogenic activity, protecting against cold challenge and high‐fat diet (HFD)‐induced obesity. Collectively, these results point to the Gabpα‐Pparγ complex as a potent regulator of glycolysis in BAT and may represent a promising therapeutic target for metabolic disease intervention.

## Graphical abstract

This study identifies the Gabpα–Pparγ complex as a key transcriptional regulator that couples glycolysis to lactate‐driven thermogenesis in brown adipose tissue by directly activating *Eno1* transcription. ‌Competitive disruption of this interaction impairs lactate metabolism and thermogenic capacity. These findings reveal a pivotal glycolysis‐lactate regulatory axis in brown fat.

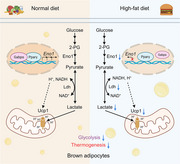

## 1 Introduction

Adipose tissues play crucial roles in the regulation of systemic metabolism and energy homeostasis.^[^ ^1^, ^2^ ^]^ To date, three distinct types of adipose tissue have been characterized: white adipose tissue (WAT), which exclusively serves as a lipid reserve; metabolically active brown adipose tissue (BAT); and inducible beige adipose tissue, which exhibits thermogenic potential under specific stimuli.^[^ ^3^, ^4^, ^5^ ^]^ BAT functions as a major “metabolic sink” for glucose and fatty acids, which contributes to the uncoupling of the respiratory chain in oxidative phosphorylation and leads to increased heat production.^[^ ^6^, ^7^ ^]^ This thermogenic activity contributes to elevated energy expenditure, thereby improving cold tolerance and combating obesity‐ related disorders.^[^ ^8^, ^9^, ^10^ ^]^ Glucose is the primary fuel source for BAT.^[^ ^11^, ^12^ ^]^ However, chronic exposure to excess nutrients leads to obesity and BAT dysfunction, a process characterized by decreased mitochondrial density, reduced thermogenic capacity, and increased triglyceride storage in BAT depots.^[^ ^13^, ^14^, ^15^ ^]^ Thus, the cellular and transcriptional dynamics of BAT maintenance evoke much attention.^[^ ^16^ ^]^

Glycolysis is an essential physiological process for the energy metabolism of BAT. In fact, functional brown fat in adults has been identified by ^18^F‐fluorodeoxyglucose positron emission tomography‐computed tomography (18F‐FDG PET‐CT),^[^ ^17^ ^]^ and cold exposure or β‐adrenergic activation promotes the uptake of glucose in BAT.^[^ ^18^ ^]^ Studies have reported that glycolysis plays an instrumental role in fueling thermogenesis.^[^ ^19^, ^20^, ^21^ ^]^ In particular, the end product of glycolysis, lactate, increases *Ucp1* mRNA levels.^[^ ^22^ ^]^ In addition, the pyruvate and lactic acid shuttle can feed into the mitochondrial tricarboxylic acid (TCA) cycle to further support free fatty acid‐dependent Ucp1‐mediated thermogenesis.^[^ ^22^ ^]^ However, the transcriptional mechanisms governing glycolysis in brown fat have yet to be identified.

Cell type specific features of brown adipocytes are regulated and determined by multiple transcription factors.^[^ ^23^, ^24^, ^25^ ^]^ Previous studies have revealed that brown adipocytes have been shown to share a developmental origin with skeletal muscle cells.^[^ ^26^, ^27^ ^]^ Specifically, PR domain‐containing 16 (Prdm16) controls the BAT/skeletal muscle switch by binding to Pparγ, enhancing the transcriptional activity of brown fat‐specific gene programs. Early B‐cell factor‐2 (Ebf2) also establishes BAT characteristics by promoting the recruitment of Pparγ to BAT‐selective target genes (including *Prdm16*).^[^ ^28^ ^]^ Moreover, Pparγ has been shown to interact with cysteine dioxygenase 1 (Cdo1) to promote the expression of lipolytic genes, facilitating energy mobilization required for thermoregulation, particularly under cold stimuli.^[^ ^29^ ^]^ Thus, Pparγ is a prerequisite for BAT development. Whether additional transcription factors cooperate with Pparγ to determine BAT identity remains to be elucidated.

Previously, the GA binding protein alpha chain (Gabpα) was identified as a key regulator of glycolytic beige adipocytes, which arise via a myogenic intermediate.^[^ ^30^, ^31^ ^]^ Notably, our study revealed that Gabpα was more highly enriched in BAT than in WAT. Specific depletion of Gabpα in BAT resulted in a white fat‐like phenotype, attenuated glycolysis and thermogenesis, and impaired cold tolerance. In contrast, specific overexpression of Gabpα in adipocytes enhanced BAT glycolysis, increased energy expenditure, and alleviated HFD‐induced obesity. Furthermore, we found that Gabpα directly bound to Pparγ to promote the transcription of the glycolytic gene *Eno1*. Disruption of Gabpα‐Pparγ binding using a competitive peptide inhibited the Eno1 expression and impaired glycolytic properties in BAT. Inhibition of Gabpα‐Pparγ complex binding also decreased lactate levels and lactate dehydrogenase (Ldh) expression, which reduced the Ucp1 abundance and impaired thermogenesis. Taken together, we identified the Gabpα‐Pparγ complex as a novel transcriptional regulator that is critical for sustaining BAT glycolytic activity and thermogenic function.

## 2 Results

### 2.1 Gabpα is Enriched in BAT and is Essential for the Glycolytic Capacity of Brown Adipocytes

**Figure 1:**
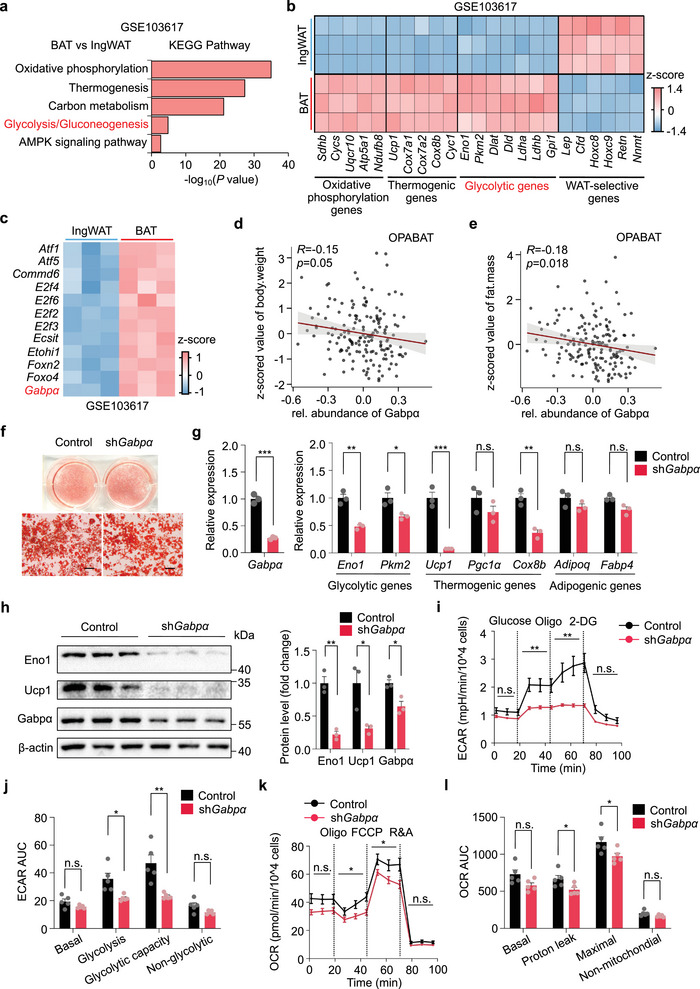
Gabpα is enriched in BAT and is essential for the glycolytic capacity of brown adipocytes. a) KEGG pathway enrichment analysis of public data for BAT versus IngWAT (GSE103617). *P* values (‐log10) according to a delta method‐based test. b) Relative mRNA levels in BAT versus IngWAT for specific marker genes in upregulated and downregulated pathways according to public data (GSE103617). c) The top DNA‐binding transcription factors in terms of molecular function according to GO analysis of BAT versus IngWAT via public data (GSE103617). d,e) Relative abundance of Gabpα and body weight (d) and fat mass (e) in 163 fully genotyped diverse outbred mice from the OPABAT website. f) Oil red O staining of differentiated iBAT‐SVF cells after *Gabpα* knockdown. Scale bar, 100 µm. g) Relative mRNA levels in differentiated iBAT‐SVF cells after *Gabpα* knockdown (*n* = 3). h) Western blot analysis of Eno1, Ucp1, and Gabpα in differentiated iBAT‐SVF cells after *Gabpα* knockdown. i–l) ECAR (i,j) and OCR (k,l) measurements in differentiated iBAT‐SVF cells after *Gabpα* knockdown (*n* = 5). The data are presented as the means ± s.e.m. *p < 0.05, **p < 0.01, and ***p < 0.001 according to a two‐tailed unpaired Student's *t* test.

Analysis of publicly available transcriptomic datasets (GSE103617) revealed that several signaling pathways and marker genes associated with oxidative phosphorylation, thermogenesis, and glycolysis were dramatically enriched in BAT compared with those in inguinal white adipose tissue (IngWAT) (**Figure** **1**a,b). Notably, Gene Ontology (GO) analysis of the top DNA‐binding transcription factors categorized by molecular function identified that *Gabpα* expression was significantly upregulated in BAT at room temperature (Figure 1c). Consistently, we found that Gabpα protein was more enriched in BAT than in IngWAT and epididymal white adipose tissue (EpiWAT) (Figure S1a, Supporting Information). Furthermore, we also detected that the mRNA levels of *Gabpα*, along with thermogenic (*Ucp1*) and glycolytic (*Eno1, Pkm2*) genes, were higher in BAT‐derived primary mature adipocytes compared to IngWAT and EpiWAT (Figure S1b, Supporting Information), consistent with the human data (Figure S1c, Supporting Information).^[^ ^32^ ^]^ Notably, using Outbred Proteomic Architecture of BAT (OPABAT),^[^ ^33^ ^]^ we found a significant negative correlation between the z‐scored value of body weight/fat mass and the relative abundance of Gabpα, indicating a potential inverse relationship between BAT Gabpα levels and obesity (Figure 1d,e). In the mouse model, the transcript level of *Gabpα* in BAT increased with cold exposure, consistent with the increased expression of thermogenic and glycolytic genes (Figure S1d, Supporting Information). Conversely, Gabpα and Eno1 protein levels were reduced in the BAT of high‐fat diet (HFD)‐fed mice compared to those fed a normal diet (Figure S1e, Supporting Information). Moreover, *Gabpα* mRNA expression was increased during differentiation of immortalized BAT (iBAT)‐derived stromal vascular fraction (SVF) cells, in parallel with increased expression of *Ucp1*, *Eno1*, *Pkm2*, and *Adipoq* expression (Figure S1f, Supporting Information). From the data of OPABAT, Gabpα expression was significantly correlated with the Pparγ, a key transcription factor involved in BAT development, but was not correlated with WAT markers such as Retn (Figure S1g,h, Supporting Information). These results indicate that Gabpα is preferentially enriched in BAT and positively associated with its metabolic and thermogenic activity.

To further investigate the functional role of *Gabpα* in brown adipocytes, we performed a knockdown of *Gabpα* in differentiated iBAT‐SVF cells via shRNA. *Gabpα* caused no morphological differences between the *Gabpα*‐knockdown group and the control group (Figure 1f). Notably, *Gabpα* knockdown significantly reduced the expression of glycolytic genes (*Eno1, Pkm2*) and thermogenic genes (*Ucp1, Cox8b*) but not of adipogenic genes (*Adipoq*, *Fabp4*) (Figure 1g,h). These results reveal that Gabpα is more critical for BAT cell function than for adipogenic differentiation. In addition, metabolic analysis revealed that both the extracellular acidification rate (ECAR) and oxygen consumption rate (OCR) were significantly reduced in differentiated iBAT‐SVF cells with *Gabpα* knockdown, indicating impaired glycolytic and mitochondrial respiratory activity (Figure 1i–l). Overall, these in vitro data indicated that Gabpα plays a pivotal role in regulating the glycolytic and thermogenic capacity of brown adipocytes.

### 2.2 Deletion of Gabpα in BAT Impairs Glycolysis and Energy Expenditure

**Figure 2:**
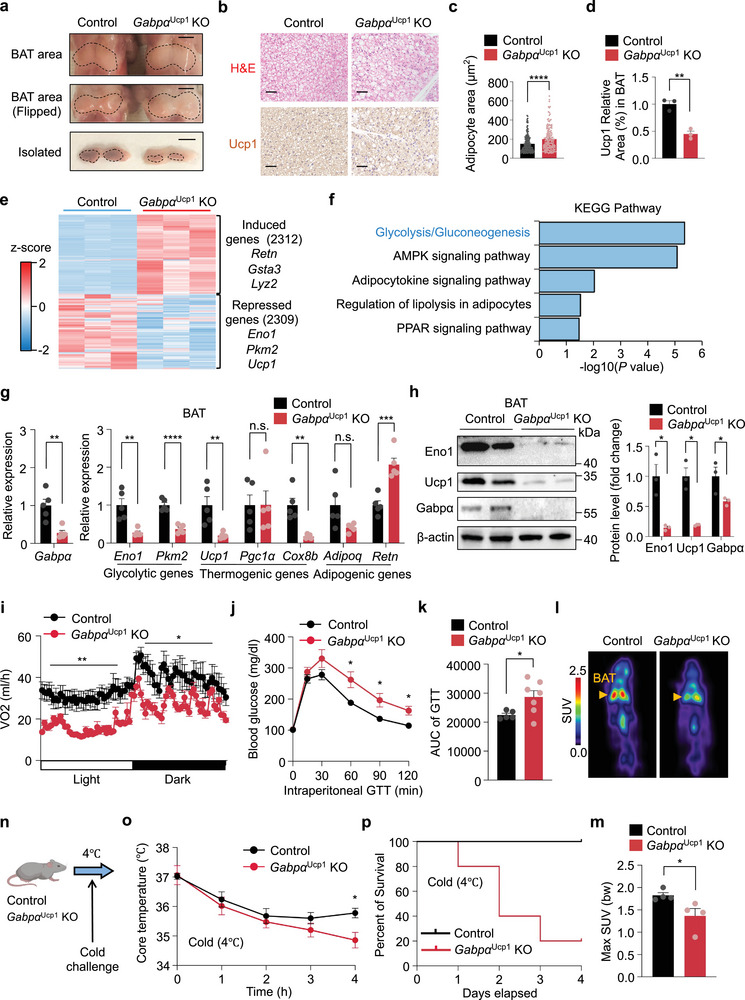
Deletion of Gabpα in BAT impairs glycolysis and energy expenditure. a) Morphology of BAT from control and *Gabpα* ^Ucp1^ KO mice (three months old) in vivo (flipped) and in the isolated state. Scale bar, 5 mm. b) H&E staining and immunostaining of Ucp1 in BAT from *Gabpα* ^Ucp1^ KO mice and littermate control mice. Scale bar, 50 µm. c) Quantification of the adipocyte area (µm^2^) in Figure 2b. d) Quantification of Ucp1 Relative area (%) in BAT in Figure 2b. e) Transcriptomic analysis of differentially upregulated and downregulated genes in BAT from control and *Gabpα* ^Ucp1^ KO mice (*n* = 3). f) Downregulated KEGG pathway enrichment analysis of BAT from control and *Gabpα* ^Ucp1^ KO mice (*n* = 3). *P* values (−log10) calculated by a delta method‐based test. g) Relative mRNA levels of related genes in BAT from control and *Gabpα* ^Ucp1^ KO mice (*n* = 5). h) Western blot analysis of Eno1, Ucp1, and Gabpα in BAT from control and *Gabpα* ^Ucp1^ KO mice. i) Oxygen consumption of control and *Gabpα* ^Ucp1^ KO mice (*n* = 4). j) Glucose tolerance test in control and *Gabpα* ^Ucp1^ KO mice (control, *n* = 5; *Gabpα* ^Ucp1^ KO, *n* = 7). k) Area under the curve (AUC) quantification of the glucose tolerance test results in Figure 2j. l,m) Max SUV of glucose in BAT through ^18^F‐FDG PET/CT of control and *Gabpα* ^Ucp1^ KO mice at room temperature (*n* = 4). n) Experimental design. Three‐month‐old control and *Gabpα* ^Ucp1^ KO mice were exposed to cold temperatures (4 °C) for cold challenge. o,p) Rectal core body temperatures (o) and percent survival (p) of *Gabpα* ^Ucp1^ KO mice and littermate control mice (*n* = 5) after cold stimulation for the indicated times at 4 °C. The data are presented as the means ± s.e.m. *p < 0.05, **p < 0.01, ***p < 0.001, and ****p < 0.0001 according to a two‐tailed unpaired Student's *t* test.

To investigate the physiological importance of Gabpα in vivo, we generated *Gabpα* ^Ucp1^ KO transgenic mice by crossing *Gabpα* ^flox/flox^ mice with Ucp1‐Cre mice (Figure S2a, Supporting Information). In *Gabpα* ^Ucp1^ KO mice, BAT displayed a WAT‐like morphology (**Figure** **2**a). Furthermore, compared to control mice (*Gabpα* ^flox/flox^), *Gabpα* ^Ucp1^ KO mice exhibited reduced Ucp1 protein levels and increased lipid accumulation in BAT, which was accompanied by a decrease in BAT mass (Figure 2a–d). However, no significant differences were observed in body weight, tissue mass, or the morphology of IngWAT, EpiWAT, or the liver between the two groups (Figure S2b–d, Supporting Information). To further investigate the effects of Gabpα loss on transcriptional programs in BAT, we performed total RNA sequencing (RNA‐seq) of BAT from *Gabpα* ^Ucp1^ KO and control mice. There were 2312 induced genes involved in WAT‐selective genes (*Retn, Gsta3*, and *Lyz2*) and 2309 downregulated genes involved in glycolysis (*Eno1, Pkm2*) and thermogenesis (*Ucp1*) (Figure 2e). Consistently, Kyoto Encyclopedia of Genes and Genomes (KEGG) pathway analysis revealed that glycolysis was significantly downregulated in *Gabpα* ^Ucp1^ KO mice (Figure 2f). Next, we identified a significant decrease in the expression of glycolytic and thermogenic genes but not of adipogenic genes in BAT from *Gabpα* ^Ucp1^ KO mice (Figure 2g,h). In contrast, there were no significant differences in the expression of genes involved in glycolysis, thermogenesis, or adipogenesis in the IngWAT of *Gabpα* ^Ucp1^ KO mice (Figure S2e, Supporting Information).

To assess the impact of Gabpα deletion on BAT function in energy metabolism, *Gabpα* ^Ucp1^ KO mice and control mice were subjected to metabolic monitoring using the Comprehensive Laboratory Animal Monitoring System (CLAMS). The data revealed that the *Gabpα* ^Ucp1^ KO mice exhibited significantly decreased oxygen consumption and heat production over a 24 h period (Figure 2i; Figure S2f–h, Supporting Information). Next, glucose tolerance tests were performed to examine glucose utilization ability in both groups of mice. Compared with control mice, *Gabpα* ^Ucp1^ KO mice exhibited impaired glucose utilization (Figure 2j,k). In the ^18^F‐FDG PET/CT experiment at room temperature (23 °C), the BAT of *Gabpα* ^Ucp1^ KO mice presented reduced glucose uptake, with a lower maximum standardized uptake value (max SUV) than that of the control mice (Figure 2l,m). In the acute cold exposure experiment (Figure 2n), the core body temperature dropped more rapidly in *Gabpα* ^Ucp1^ KO mice than in controls, indicating impaired cold tolerance (Figure 2o). Moreover, *Gabpα* ^Ucp1^ KO mice exhibited increased mortality during a 4‐day cold exposure (Figure 2p). Collectively, these results suggest that Gabpα is indispensable for maintaining glycolytic function and energy expenditure in brown fat.

### 2.3 Gabpα Binds to Pparγ to Increase the Transcription of *Eno1*

**Figure 3:**
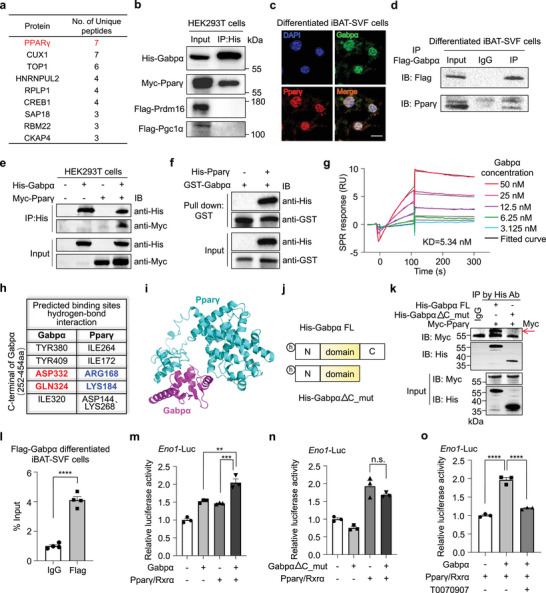
Gabpα binds to Pparγ to increase the transcription of *Eno1*. a) Immunoprecipitated by Flag and IgG antibody for protein capture by LC‐MS/MS in HEK293T cells infected with GABPα‐Flag overexpression plasmid. List of the top candidate proteins pulled down by GABPα when the fold change of intensity was greater than 3 between the Flag‐GABPα and IgG groups. b) Immunoblotting for various brown fat‐specific transcription factors after IP with a anti‐His antibody in HEK293T cells cotransfected with Gabpα and the indicated transcription factors. IP, immunoprecipitation. c) Immunofluorescence analysis of the colocalization of Gabpα and Pparγ in the nuclei of differentiated iBAT‐SVF cells. Scale bar, 10 µm. d,e) Co‐IP was used to validate the physical interaction between the Gabpα and Pparγ proteins in cell lysates from differentiated iBAT‐SVF cells (d) transfected with Flag‐Gabpα overexpressing lentivirus and in HEK293T cells (e) transfected with the indicated plasmids. IP, immunoprecipitation. f) In vitro binding assay of Gabpα‐GST and purified Pparγ fragments. g) SPR sensorgrams showing the interaction between different concentrations of Gabpα and Pparγ protein. h) Five amino acids of Gabpα and six amino acids of Pparγ were predicted to bind via hydrogen bonds. The labeled portions are the adjacent amino acid binding sites. i) Protein docking site prediction of Gabpα and Pparγ via the HDOCK server. j) Schematic illustration of C‐terminal truncated mutants of Gabpα. N, N‐terminal; C, C‐terminal; h, His‐tagged. k) Identification of the Gabpα‐binding domain in Pparγ. HEK293T cells were cotransfected with Myc‐Pparγ and His‐Gabpα‐FL or with C‐terminal truncation mutants of Gabpα. IP was performed with anti‐His antibodies. IP, immunoprecipitation. l) RT‐qPCR quantifying the amount of immunoprecipitated DNA containing the putative Gabpα binding site located in the *Eno1* using the FLAG compared to IgG antibody in Flag‐Gabpα overexpressed differentiated iBAT‐SVF cells. m,n) Luciferase reporter assay showing the transcriptional activity of *Eno1*‐Luc by the Gabpα (m) or C‐terminal truncation mutants of Gabpα (n) and Pparγ/Rxrα proteins in HEK293T cells. *n* = 3 biologically independent samples. o) Luciferase reporter assay showing the transcriptional activity of *Eno1*‐Luc by the Gabpα and Pparγ/Rxrα proteins in HEK293T cells treated with T0070907 (10 µm). *n* = 3 biologically independent samples. The data are presented as the means ± s.e.m. **p < 0.01, ***p < 0.001, and ****p < 0.0001 according to a two‐tailed unpaired Student's *t* test for comparisons between two groups and one‐way ANOVA followed by Tukey's test for comparisons among multiple groups.

To identify the potential cofactor interacting with Gabpα and regulating the glycolytic function of BAT, we performed an immunoprecipitation of Gabpα and subjected all associated proteins to liquid chromatography‐tandem mass spectrometry (LC/MS‐MS) analysis. Interestingly, the results pinpointed that Pparγ, a key regulator of BAT development, was the most promising candidate protein that interacts with Gabpα when the fold change of intensity was greater than 3 between the Flag‐Gabpα and IgG groups (**Figure** **3**a; Figure S3a, Supporting Information). We further tested whether Gabpα interacts with other key transcriptional factors known to orchestrate thermogenic programming in BAT, such as Prdm16 and Pgc1α.^[^ ^24^, ^26^, ^34^, ^35^ ^]^ To confirm these interactions, we then co‐transfected Myc‐Pparγ, Flag‐Pgc1α, Flag‐Prdm16, and His‐Gabpα into HEK293T cells, and immunoprecipitated Gabpα with an anti‐His antibody. The results unequivocally demonstrated that Gabpα clearly interacted with Pparγ but not with Prdm16 or Pgc1α (Figure 3b). We further verified that the interaction occurred only between Gabpα and Pparγ via co‐immunoprecipitation (Co‐IP) assays (Figure S3b–d, Supporting Information). Moreover, immunofluorescence analysis revealed colocalization of Gabpα and Pparγ within the nuclei of differentiated iBAT‐SVF cells (Figure 3c). In support of these findings, Gabpα interacted with Pparγ in differentiated iBAT‐SVF cells and in HEK293T cells (Figure 3d,e). Notably, a glutathione S‐transferase (GST)‐tagged full‐length Gabpα protein was able to pull down Pparγ in vitro, which indicated the existence of a direct interaction between these two proteins (Figure 3f). In addition, the response signal intensity between Gabpα and Pparγ in the Surface Plasmon resonance (SPR) sensorgram increased with the concentration of Gabpα (Figure 3g), with a binding constant (*K*_D_) of 5.34 nm, indicating a strong binding affinity between the two proteins.

To further explore the binding sites of Gabpα and Pparγ, we visualized the molecular structures of these two proteins by using the Protein Data Bank (PDB) and then predicted the binding sites by using HDOCK, a web server for protein‐protein and protein‐DNA/RNA docking based on a hybrid algorithm (Figure 3h,i; Figure S3e, Supporting Information).^[^ ^36^ ^]^ The docking site map revealed that five amino acids in Gabpα formed hydrogen bonds with six amino acids in Pparγ, with a calculated protein docking binding energy of −248.52 kcal mol^−1^. Notably, all five binding residues in Gabpα were localized within its C‐terminus region (aa 252–454). To investigate whether these binding sites are essential for binding, we constructed C‐terminal truncation mutants of His‐tagged Gabpα, as shown in Figure 3j. The binding sites necessary to interact with Pparγ were confirmed to be located at the C‐terminus of Gabpα (Figure 3k).

We next examined whether the Gabpα‐Pparγ complex affects the transcriptional regulation of glycolytic genes in brown fat. Because Gabpα loss substantially repressed the glycolysis and decreased the expression of glycolytic genes, we focused on the transcriptional regulatory role of Gabpα‐Pparγ complex in this process. Based on the canonical Gabpα DNA‐binding motif retrieved from the JASPAR database (Figure S3f, Supporting Information), we scanned the proximal promoter region of the glycolytic gene *Eno1*. A putative Gabpα binding site was identified 1505 bp upstream of the *Eno1* transcription start point (Figure S3g, Supporting Information). Next, we performed chromatin immunoprecipitation (ChIP) studies on the putative Gabpα binding site in the *Eno1* gene and found enriched occupancy at that site in differentiated iBAT‐SVF cells overexpressing Flag‐tagged Gabpα (Figure 3l). To assess the transcriptional activity of this region, we subcloned the *Eno1* promoter and its upstream sequence, which contains this potential Gabpα binding site, into a promoterless luciferase reporter construct. Luciferase reporter assays revealed that Gabpα alone enhanced *Eno1* promoter activity. Notably, co‐transfection of Gabpα and Pparγ further increased *Eno1*‐driven luciferase activity, which indicates the synergetic transcriptional regulation of *Eno1* by the Gabpα‐Pparγ complex (Figure 3m). However, this cooperative activation was abolished when in Gabpα C‐terminal truncation mutants lacking the Pparγ‐binding domain (Figure 3n). Furthermore, the *Eno1* promoter activity induced by Gabpα could be inhibited via T0070907, a potent and selective Pparγ antagonist (Figure 3o). Collectively, these data suggest that Gabpα directly interacts with Pparγ to form a transcriptional complex that coordinatively regulates the expression of glycolytic gene *Eno1*. This regulatory mechanism likely contributes to the glycolytic function and molecular identity of brown adipocytes

### 2.4 Inhibition of Gabpα‐Pparγ Binding Represses Glycolytic Capacity in BAT

**Figure 4:**
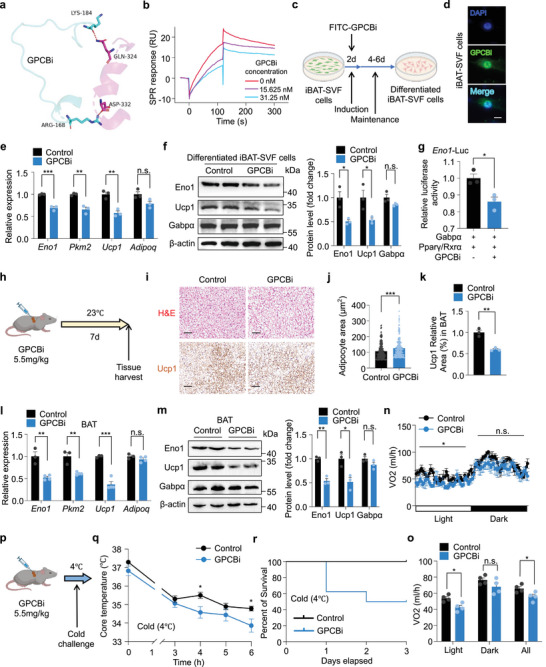
Inhibition of Gabpα‐Pparγ binding represses glycolytic capacity in BAT. a) Schematic illustration of the peptide GPCBi designed for competitive binding to adjacent amino acid binding sites of Gabpα. b) SPR sensorgrams showing the influence of GPCBi on Gabpα and Pparγ interaction. c) Experimental design. Intervention of the peptide FITC‐GPCBi (5 µm) on iBAT‐SVF cells during the first 2 days of adipogenic differentiation. d) Immunofluorescence analysis of the colocalization of FITC‐GPCBi and DAPI in the nuclei of iBAT‐SVF cells. Scale bar, 10 µm. e,f) Relative mRNA (e) (n = 3) and protein (f) levels in differentiated iBAT‐SVF cells after intervention with the peptide GPCBi (5 µm) during the first 2 days of adipogenic differentiation. g) Luciferase reporter assay showing the transcriptional activity of *Eno1*‐Luc by the Gabpα and Pparγ/Rxrα proteins in HEK293T cells treated with GPCBi (3 µm). *n* = 3 biologically independent samples. h) Experimental design. Three‐month‐old wild‐type mice were orthotopically injected with the peptide GPCBi (5.5 mg kg^−1^) or scramble peptide in BAT and sacrificed after 7 days. i) H&E staining and immunostaining of Ucp1 in BAT from wild‐type mice treated with the peptide GPCBi. Scale bar, H&E staining, 50 µm; immunostaining of Ucp1, 100 µm. j) Quantification of the adipocyte area (µm^2^) in Figure 4i. k) Quantification of Ucp1 Relative area (%) in BAT in Figure 4i. l,m) Relative mRNA (l) (control, *n* = 3; GPCBi, *n* = 4) and protein (m) levels in BAT from wild‐type mice treated with the peptide GPCBi. n,o) Oxygen consumption of wild‐type mice treated with the peptide GPCBi (*n* = 4). p) Experimental design. Wild‐type mice treated with the peptide GPCBi were exposed to cold temperatures (4 °C) for cold challenge. q,r) Rectal core body temperatures (q) and percent survival (r) of wild‐type mice treated with the peptide GPCBi (control, *n* = 7; GPCBi, *n* = 8) upon cold stimulation at 4 °C for the indicated times. The data are presented as the means ± s.e.m. *p < 0.05, **p < 0.01, and ***p < 0.001 according to a two‐tailed unpaired Student's *t* test.

Since the protein levels of Gabpα and Eno1 were decreased in BAT of HFD mice (Figure S1e, Supporting Information), we then explored the binding dynamics of Gabpα‐Pparγ transcriptional complex. Notably, there was a weakened interaction between Gabpα and Pparγ in brown fat from HFD mice, which may explain the observed downregulation of Eno1 (Figure S4a,b, Supporting Information). To mimic the disrupted binding state, we designed a synthetic polypeptide containing the same amino acids near the binding sites in Pparγ (aa 164–189), including ARG168 and LYS184, which competitively bind to Gabpα at ASP332 and GLN324, respectively (**Figures** **4**a and 3h). This peptide, termed Gabpα‐Pparγ complex binding inhibitor (GPCBi), was tested for its ability to competitively block Gabpα–Pparγ interaction. We performed a Co‐IP assay and found that the binding capacity between Gabpα and Pparγ decreased when GPCBi was added (Figure S4c, Supporting Information). Moreover, GPCBi was proven to have a direct binding with Gabpα in a dose‐dependent manner, and the *K*_D_ value is 7.03 nm (Figure S4d, Supporting Information). In the absence of GPCBi, Gabpα showed a high affinity toward Pparγ, whereas GPCBi treatment dose‐dependently attenuated this interaction (Figure 4b). These data confirmed the competitive binding of GPCBi on Gabpα‐Pparγ complex.

Next, we explored the effect of GPCBi on differentiated iBAT‐SVF cells in vitro (Figure 4c). We first verified the nuclear localization of FITC‐tagged GPCBi in iBAT‐SVF cells (Figure 4d). Supplying GPCBi to differentiated iBAT‐SVF cells decreased the expression of glycolytic and thermogenic genes but did not change the expression of adipogenic gene *Adipoq* (Figure 4e,f). Consistently, luciferase reporter assays showed that GPCBi suppressed the Gabpα‐Pparγ complex‐mediated activation of the *Eno1* promoter (Figure 4g). We next asked whether inhibiting the formation of the Gabpα‐Pparγ transcriptional complex in vivo would similarly impair BAT function. To test this hypothesis, we orthotopically injected GPCBi or scramble peptide into the BAT of wild‐type mice at room temperature and harvested the tissues after one week (Figure 4h). GPCBi‐treated mice exhibited morphological changes and reduced BAT mass, whereas no significant changes in body weight or the morphology or tissue mass of IngWAT, EpiWAT, or the liver between these mouse groups (Figure S4e–g, Supporting Information). Furthermore, GPCBi injection increased lipid accumulation and decreased Ucp1 abundance in BAT (Figure 4i–k). The gene expressions of *Eno1*, *Pkm2*, and *Ucp1* in BAT also decreased with the intervention of GPCBi (Figure 4l,m). To evaluate the systemic metabolic consequences of inhibiting the Gabpα‐Pparγ complex, we measured metabolic parameters in GPCBi‐injected mice. The mice given GPCBi presented markedly increased fasting blood glucose levels (Figure S4h, Supporting Information), along with significant decreases in oxygen consumption and heat production at room temperature (Figure 4n,o; Figure S4i,j, Supporting Information). Consistent with these findings, GPCBi‐treated mice struggled to maintain their core body temperatures under acute cold stress conditions, resulting in increased mortality (Figure 4p–r). These results indicate that the Gabpα‐Pparγ transcriptional complex is imperative for sustaining BAT capacity and systemic metabolic homeostasis.

### 2.5 Disruption of Gabpα‐Pparγ Complex Formation Suppresses Ucp1 Expression via Decreased Lactate Levels

**Figure 5:**
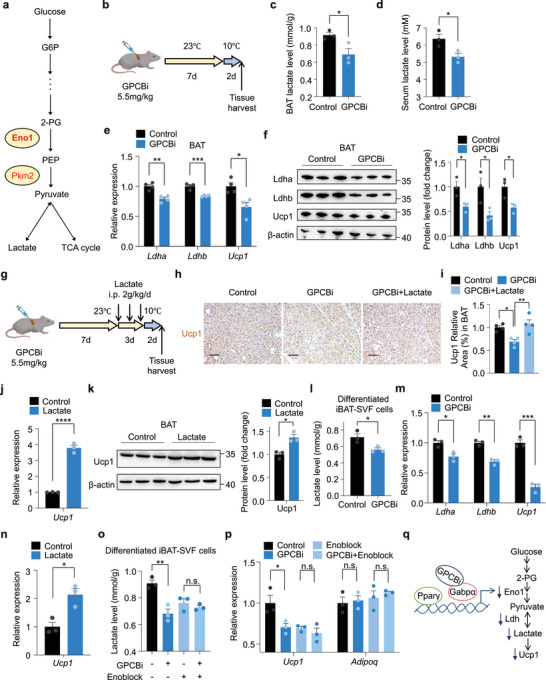
Blockade of Gabpα‐Pparγ complex binding impairs Ucp1 expression via decreased lactate levels. a) Schematic diagram of the process of glycolysis with the pivotal regulatory enzymes Eno1 and Pkm2. b) Experimental design. Three‐month‐old wild‐type mice were orthotopically injected with the peptide GPCBi (5.5 mg kg^−1^) or scramble peptide in BAT at 23 °C for 7 days and then at 10 °C for 2 days. c,d) Lactate levels in BAT (c) and serum (d) from wild‐type mice treated with the peptide GPCBi at 23 °C for 7 days to 10 °C for 2 days (*n* = 3). e) Relative mRNA levels of *Ldha*, *Ldhb*, and *Ucp1* in BAT from wild‐type mice treated with the peptide GPCBi at 23 °C for 7 days to 10 °C for 2 days (*n* = 4). f) Western blot analysis of Ldha, Ldhb, and Ucp1 in BAT from wild‐type mice treated with the peptide GPCBi at 23 °C for 7 days to 10 °C for 2 days. g) Experimental design. Three‐month‐old wild‐type mice were orthotopically injected with the peptide GPCBi (5.5 mg kg^−1^) in BAT for 7 days and then intraperitoneally injected with sodium lactate (2 g kg^−1^ d^−1^) daily for 3 days following 10 °C for 2 days. h) Immunostaining of Ucp1 in BAT from the wild‐type mouse model shown in Figure 5g. Scale bar, 100 µm. i) Quantification of Ucp1 Relative area (%) in BAT in Figure 5h. j,k) Relative mRNA (j) (*n* = 3) and protein (k) levels in BAT from wild‐type mice treated with intraperitoneally injected sodium lactate (2 g kg^−1^ d^−1^) daily for 3 days. l) Lactate levels in differentiated iBAT‐SVF cells after intervention with the peptide GPCBi (5 µm) during the first 2 days of adipogenic differentiation. m) Relative mRNA levels of *Ldha*, *Ldhb*, and *Ucp1* in differentiated iBAT‐SVF cells after intervention with the peptide GPCBi (5 µm) during the first 2 days of adipogenic differentiation. n) Relative *Ucp1* mRNA levels in differentiated iBAT‐SVF cells after intervention with sodium lactate (30 mm) for 24 h. o) Lactate levels in differentiated iBAT‐SVF cells after intervention with the peptide GPCBi (5 µm) during the first 2 days of adipogenic differentiation and Enoblock (10 µm) for 24 h. p) Relative *Ucp1* and *Adipoq* mRNA levels in differentiated iBAT‐SVF cells after intervention with the peptide GPCBi (5 µm) during the first 2 days of adipogenic differentiation and Enoblock (10 µm) for 24 h. q) Schematic diagram. Inhibition of Gabpα‐Pparγ binding represses the transcription of *Eno1*, which further impairs Ucp1 expression via decreased lactate levels. The data are presented as the means ± s.e.m. *p < 0.05, **p < 0.01, ***p < 0.001, and ****p < 0.0001 according to a two‐tailed unpaired Student's *t* test for comparisons between two groups and one‐way ANOVA followed by Tukey's test for comparisons among multiple groups.

Inhibition of Gabpα‐Pparγ binding substantially repressed the expression of Eno1 and Pkm2, two key glycolytic enzymes (**Figure** **5**a). We further investigated whether the intervention of GPCBi in brown fat alters lactate levels, the end product of glycolysis. After being subjected to room temperature for 7 days and moderate cold exposure (10 °C) for 2 days (Figure 5b), the GPCBi intervention substantially suppressed the production of lactate in brown fat and in circulation (Figure 5c,d). Given that previous reports have shown that lactate can increase Ucp1 levels via lactate utilization by lactate dehydrogenase (Ldh),^[^ ^37^, ^38^ ^]^ we proceeded to assess the role of GPCBi in the expression of Ldh. Indeed, GPCBi effectively downregulated both Ldh and Ucp1 expression in BAT (Figure 5e,f). However, exogenous lactate supplementation rescued the Ucp1 expression suppressed by GPCBi (Figure 5g–i). Moreover, lactate itself increased Ucp1 expression in brown fat (Figure 5j,k), consistent with the results reported previously.^[^ ^22^, ^39^ ^]^ These findings were further supported by ex vivo experiments. Inhibition of Gabpα‐Pparγ complex formation decreased intracellular lactate levels in brown adipocytes, along with the lower expression of Ldh and Ucp1 (Figure 5l,m). Conversely, treatment for 24 h with lactate triggered Ucp1 expression in brown adipocytes (Figure 5n). To further identify GPCBi‐induced decrease in lactate and impaired downstream signaling of thermogenesis are dependent on Eno1. We used the Enoblock, a pharmacological inhibitor of Eno1, in GPCBi‐treated differentiated iBAT‐SVF cells. Lactate level was reduced in GPCBi‐treated brown adipocytes than the scramble peptide‐treated group, however, this phenomenon was not observed in Enoblock‐treated cells. Consistently, the thermogenic gene *ucp1* were downregulated in GPCBi‐treated brown adipocytes, but remained no change when further added Enoblock. These results indicate that Eno1 is a molecular link between Gabpα‐Pparγ complex and lactate‐dependent thermogenesis in brown fat (Figure 5o,p). These findings suggest that disruption of Gabpα‐Pparγ binding impairs Ucp1 expression by perturbing glycolytic gene Eno1 and lactate metabolism (Figure 5q). Emerging studies indicate Ucp1‐independent thermogenesis are ATP‐linked substrate futile cycles. We next investigate the role of interventions with GPCBi and lactate on these futile cycles in brown adipocytes in vivo and in vitro, including the futile creatine cycle, calcium cycle, and lipid cycle.^[^ ^40^, ^41^, ^42^ ^]^ Our data revealed that GPCBi‐induced decreased glycolytic flux substantially impaired the regulatory gene expression of the futile lipid cycle, while lactate supplementation triggered the expression of these genes (Figure S5a–d, Supporting Information). These results suggest that Gabpα‐Pparγ complex‐mediated glycolytic flux and lactate production tend to be required to drive the futile lipid cycle.

### 2.6 Adipocyte‐Specific Gabpα Overexpression Enhances Glycolytic Capacity in BAT

**Figure 6:**
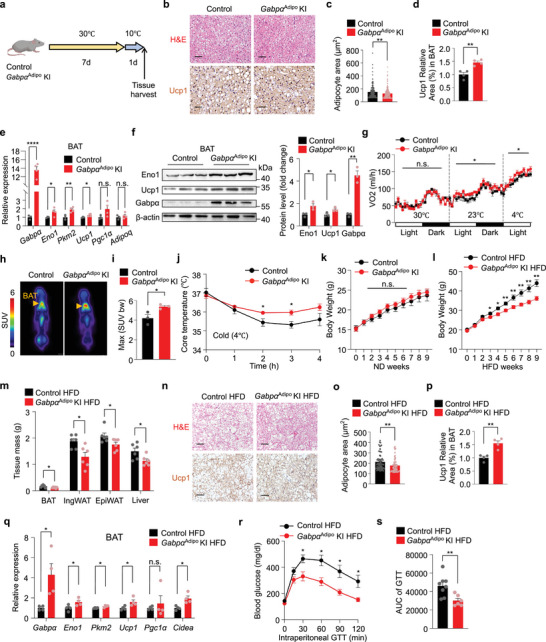
Adipocyte‐specific Gabpα overexpression enhances glycolytic capacity in BAT. a) Schematic diagram of the *Gabpα* ^Adipo^ KI mouse model (30 °C for 7 days to 10 °C for 1 day). b) H&E staining and immunostaining of Ucp1 in BAT from control and *Gabpα* ^Adipo^ KI mice after switching from 30 °C for 7 days to 10 °C for 1 day. Scale bar, H&E staining, 100 µm; immunostaining of Ucp1, 50 µm. c) Quantification of the adipocyte area (µm^2^) in Figure 6b. d) Quantification of Ucp1 Relative area (%) in BAT in Figure 6b. e,f) Relative mRNA (control, n = 3; *Gabpα* ^Adipo^ KI, n = 4) (e) and protein (f) levels in BAT from control and *Gabpα* ^Adipo^ KI mice after a switch from 30 °C for 7 days to 10 °C for 1 day. g) Oxygen consumption of control and *Gabpα* ^Adipo^ KI mice housed at a temperature gradient from 30 to 23 °C and further to 4 °C (*n* = 4). h,i) Max SUV of glucose in BAT through ^18^F‐FDG PET/CT of control and *Gabpα* ^Adipo^ KI mice after a switch from 30 °C for 7 days to 10 °C for 1 day (*n* = 3). j) Rectal core body temperatures of *Gabpα* ^Adipo^ KI mice and littermate control mice (*n* = 7) after cold stimulation for the indicated times at 4 °C. k) Weekly body weights of *Gabpα* ^Adipo^ KI mice and control mice fed a normal diet (*n* = 5). l) Weekly body weights of *Gabpα* ^Adipo^ KI mice and control mice fed a HFD (*n* = 6). m) Tissue masses of BAT, IngWAT, EpiWAT, and liver from control and *Gabpα* ^Adipo^ KI mice after a twelve‐week HFD (*n* = 6). n) H&E staining and immunostaining of Ucp1 in BAT from *Gabpα* ^Adipo^ KI mice and control mice after a twelve‐week HFD. Scale bar, H&E staining, 100 µm; immunostaining of Ucp1, 50 µm. o) Quantification of the adipocyte area (µm^2^) in Figure 6n. p) Quantification of Ucp1 Relative area (%) in BAT in Figure 6n. q) Relative mRNA levels of related genes in BAT from control and *Gabpα* ^Adipo^ KI mice after a twelve‐week HFD (*n* = 4). r) Glucose tolerance test of control and *Gabpα* ^Adipo^ KI mice after a twelve‐week HFD (control, *n* = 8; *Gabpα* ^Adipo^ KI, *n* = 6). s) Area under the curve (AUC) quantification of the glucose tolerance test results in Figure 6r. The data are presented as the means ± s.e.m. *p < 0.05, **p < 0.01, and ****p < 0.0001 according to a two‐tailed unpaired Student's *t* test.

To further assess the functional role of Gabpα in BAT, we generated *Gabpα* ^Adipo^ knock‐in (KI) mice with specific overexpression of *Gabpα* in adipocytes (Figure S6a, Supporting Information). Compared with control mice, these mice displayed increased Ucp1 expression and decreased lipid accumulation in BAT after 7 days of thermoneutrality and 1 day of moderate cold exposure (10 °C) (**Figure** **6**a–d; Figure S6b, Supporting Information). Moreover, the expression of glycolytic and thermogenic genes was markedly elevated in BAT, while remained unchanged in IngWAT from *Gabpα* ^Adipo^ KI mice under cold conditions (Figure 6e,f; Figure S6c, Supporting Information). Under thermoneutral conditions, no significant differences in either the oxygen consumption rate or heat generation between *Gabpα* ^Adipo^ KI mice and control mice. When exposed to 23 °C and subsequently to 4 °C, *Gabpα* ^Adipo^ KI mice consistently displayed greater increases in the oxygen consumption rate and heat generation compared to control mice (Figure 6g; Figure S6d–f, Supporting Information). Moreover, the *Gabpα* ^Adipo^ KI mice displayed greater BAT activity with a higher maximum SUV of glucose, as detected through ^18^F‐FDG PET/CT (Figure 6h,i). In addition, *Gabpα* ^Adipo^ KI mice increased core body temperature than littermate control mice under acute cold stress (Figure 6j). Overall, these data reveal that Gabpα functions as a crucial positive regulator of BAT function in cold‐exposed mice.

Next, we examined whether the overexpression of Gabpα in fat tissues could influence whole‐body metabolism. After 9 weeks on a normal chow diet, *Gabpα* ^Adipo^ KI mice exhibited modestly improved glucose tolerance but showed no significant difference in body weight compared to control mice (Figure 6k; Figure S7a,b, Supporting Information). Of note, following a 9‐week HFD regimen, *Gabpα* ^Adipo^ KI mice were protected against HFD‐induced obesity, exhibiting reduced weights of BAT, IngWAT, EpiWAT, and liver, despite similar food intake (Figure 6l,m; Figure S7c–f, Supporting Information). Moreover, *Gabpα* ^Adipo^ KI mice presented less lipid accumulation in BAT, IngWAT, and EpiWAT and more Ucp1 abundance in BAT than control mice in response to a HFD (Figure 6n–p; Figure S7g, Supporting Information). Additionally, we found that the expression of glycolysis and thermogenesis‐related genes was elevated in BAT but remained unchanged in the IngWAT of *Gabpα* ^Adipo^ KI mice compared to controls (Figure 6q; Figure S7h, Supporting Information). Compared with control mice, *Gabpα* ^Adipo^ KI mice presented improved glucose tolerance (Figure 6r,s) and significantly ameliorated liver steatosis (Figure S7i, Supporting Information). These results demonstrate that adipocyte‐specific overexpression of Gabpα confers protection against both cold‐induced stress and HFD‐induced obesity by promoting BAT function and systemic metabolic health.

## 3 Discussion

Targeting thermogenic adipose tissues has emerged as a promising therapeutic strategy to counteract obesity and associated metabolic diseases. BAT is a classic thermogenic fat depot, and sustaining its development and function is critical. Previous studies have shown that brown adipocytes originate from Myogenic factor 5 (Myf5)‐positive myogenic precursor cells and that this process is controlled by Prdm16.^[^ ^26^ ^]^ Additional factors, including Pparγ and Ehmt1, interact with Prdm16 to regulate BAT development and function.^[^ ^26^, ^43^, ^44^, ^45^ ^]^ A noncanonical subset of beige adipocytes, which originate from the myogenic lineage and display enhanced glucose oxidation, is referred to as glycolytic beige adipocytes.^[^ ^30^ ^]^ Notably, Gabpα, a known transcription factor of the ETS family,^[^ ^46^, ^47^ ^]^ was identified as a critical regulator of the identity of these glycolytic beige adipocytes. Given the shared myogenic origin and robust glycolytic capacity of brown adipocytes, we hypothesized that Gabpα would likewise regulate the brown adipocytes physiology. Here, we show that a Gabpα‐Pparγ complex functions as a pivotal transcriptional module that drives the glycolytic program in brown adipocytes. Importantly, guided by docking predictions, we demonstrate a direct interaction between Gabpα and Pparγ that promotes the transcription of glycolytic gene *Eno1*. Disruption of the Gabpα‐Pparγ complex in brown fat downregulated glycolytic gene as well as Ucp1 expression in addition to lactate production. Conversely, adipose‐specific Gabpα overexpression enhanced glycolysis and thermogenesis in BAT and protected against HFD‐induced obesity. These findings position the Gabpα–Pparγ interaction as a potential therapeutic target to enhance brown fat function and support systemic metabolic homeostasis.

Knockdown of *Gabpα* or disruption of Gabpα‐Pparγ binding in primary differentiated iBAT‐SVF cells reduced the expression of glycolytic and thermogenic genes, whereas adipogenic genes expression remained unchanged. These observations indicate that Gabpα–Pparγ interaction is required to sustain brown adipocyte functional identity, whereas disruption of this interaction is associated with a whitening phenotype in BAT. Previous studies have shown that the overexpression of Pparγ in adipocytes promotes the expression of thermogenic genes in BAT,^[^ ^4^ ^]^ an effect that may be further augmented by the promotion of Gabpα‐Pparγ binding. Conversely, chronic overnutrition progressively leads to obesity and is associated with the whitening of BAT in mice.^[^ ^13^ ^]^ Consistently, we observed a weakened interaction between Gabpα and Pparγ and reduced Eno1 expression in BAT of HFD mice. In fact, both Gabpα and Pparγ protein levels in BAT were markedly decreased when subjected to HFD.^[^ ^48^ ^]^ Together, these results indicate that overnutrition suppresses both the expression and interaction of Gabpα‐Pparγ complex, thereby attenuating *Eno1* transcription, impairing glycolytic capacity in BAT, and exacerbating obesity risk. Importantly, Gabpα overexpression reversed HFD‐induced BAT dysfunction and attenuated diet‐induced obesity. Therefore, Gabpα, through its interaction with Pparγ, supports BAT glycolytic function and metabolic health, particularly under nutritional excess.

In our study, we reveal that the Gabpα‐Pparγ complex coordinates the transcriptional program of glycolysis by activating *Eno1*. Pharmacologic disruption of Gabpα‐Pparγ binding with GPCBi lowered lactate levels in BAT and circulation, accompanied by reduced *Ldh* and *Ucp1* expression and diminished thermogenic output. Consistent with prior reports that brown fat glycolysis is critical for Ucp1‐mediated thermogenesis,^[^ ^19^, ^21^ ^]^ and the glycolytic end product lactate itself increases *Ucp1* mRNA levels,^[^ ^22^, ^39^ ^]^ our results support a lactate‐dependent link between glycolytic flux and the thermogenic machinery. Mechanistically, Ldh regulates the conversion of lactate and pyruvate, which tunes cellular redox state.^[^ ^37^ ^]^ Lactate and pyruvate shuttle into mitochondria and feed into the TCA cycle,^[^ ^49^ ^]^ generating reducing equivalents that fuel mitochondrial complex I‐driven respiration and sustain the proton gradient that enables Ucp1‐mediated uncoupling. In parallel, the citrate exported from the TCA cycle supports de novo lipogenesis and then increases Ucp1 uncoupling in a free fatty acid‐dependent manner.^[^ ^50^ ^]^ In line with this model, exogenous lactate rescued the GPCBi‐induced suppression of Ucp1 expression and, on its own, increased *Ucp1* expression in BAT, consistent with previous observations. Together, these findings indicate that the Gabpα–Pparγ interaction is a pivotal node coupling glycolysis to lactate shuttle and Ucp1‐dependent thermogenesis, with *Eno1* functioning as a core glycolytic component under its transcriptional control. Interestingly, Gabpα‐Pparγ complex‐mediated glycolytic flux and lactate generation at least drive the futile lipid cycle. To further identify the effect and investigate the role on other futile cycles would depend on the Ucp1‐KO mouse model.

As previously described, Prdm16 has been shown to control BAT development. However, *Prdm16* ^adipo^ KO mice display no functional deficiencies in thermogenesis, O_2_ consumption, or glucose uptake in BAT,^[^ ^51^ ^]^ suggesting that other transcriptional pathways can sustain BAT function in its absence. Our study suggests that Gabpα acts through a distinct mechanism, independent of Prdm16 regulation. Previous work has shown that Pparγ is essential for the maintenance of BAT characteristics, since *Pparγ* ^Ucp1^ KO mice display obvious whitening in BAT.^[^ ^52^, ^53^ ^]^ This phenotype may also be attributed to a disruption of the direct binding between Gabpα and Pparγ that we observed in our study. Notably, while Gabpα binds to Pparγ, we did not detect interactions between Gabpα and either Prdm16 or Pgc1α. Consistent with this, Gabpα‐specific binding sites overlap substantially with Pparγ1‐specific sites in adipose tissue,^[^ ^54^ ^]^ suggesting a potential synergistic effect between Gabpα and Pparγ in transcriptional control. In summary, our work uncovers a previously unrecognized transcriptional regulatory mechanisms that modulates the glycolytic capacity of brown fat, thereby contributing to energy balance and tissue homeostasis. Further research is needed to identify additional factors that may regulate the formation of the Gabpα‐Pparγ complex. Additionally, while our work focused on the effects of the Gabpα‐Pparγ complex on BAT properties, we also applied this regulatory framework to induce a BAT phenotype in WAT to combat obesity and concomitant disorders.

## 4 Experimental Section

### Animals

The mouse experiments were approved by the Institutional Animal Care and Use Committee of the Institute of Model Animals of Tongji Hospital, Huazhong University of Science and Technology, and were performed in compliance with all relevant ethical regulations (approval number: TJH‐202110044). Unless otherwise noted, the mice were housed in a pathogen‐free and climate‐controlled environment (22–25 °C, 40‐60% humidity) and were given free access to food and water on a 12 h light/dark cycle. Wild‐type C57BL/6J mice were obtained from Beijing Huafukang Bioscience Co., Ltd. (Beijing, China). *Gabpa* ^flox/flox^ *, Adipoq^Cre^*, and *Ucp1^Cre^* mice were purchased from Cyagen Biosciences, Inc. (Guangzhou, China). For GPCBi treatment, wild‐type mice were orthotopically injected GPCBi (FITC‐YGRKKRRQRRRRRTIRLKLIYDRCDLNCRIHKKSRNK; 5.5 mg kg^−1^) or scramble peptide (FITC‐YGRKKRRQRRRYTNDKIRKNCRKLDRCRKILIHSRLR; 5.5 mg kg^−1^) into the BAT at room temperature for one week. For sodium lactate (MCE, Cat# HY‐B2227B) treatment, the mice received a continuous intraperitoneal injection for 3 days (2 g kg^−1^ body weight). For cold exposure, the mice were caged individually and exposed to a temperature of 10 or 4 °C. The mice were euthanized by CO_2_ inhalation. For studies with specific diets, five‐week‐old male *Gabpα* ^Adipo^ KI mice and their respective littermate control mice were fed a HFD (D12492, Research Diets; 60 kcal% fat, 20 kcal% carbohydrates, and 20 kcal% protein; Medicience Ltd., Jiangsu, China) at ambient temperature. The food was changed every week. Body weight was measured every week, and tissue weights were measured at the time of euthanasia.

### Intraperitoneal Glucose Tolerance Tests

For the glucose tolerance test, the mice were fasted for 16 h and then injected intraperitoneally with glucose (2 g kg^−1^). Tail blood samples were collected at 0, 15, 30, 60, 90, and 120 min after injection via a glucometer.

### Cell Culture

Interscapular BAT‐SVF cells were obtained from eight‐week‐old male C57BL/6 mice. Briefly, BAT was excised, washed in PBS, minced, and digested at 37 °C for 40 min in Hanks solution containing type II collagenase (Sigma, Cat# C‐6885) and 4% bovine serum albumin (BSA). The tissue homogenates were filtered through a 70 µm cell strainer and centrifuged at 1000 rpm for 5 min. The cell pellet was resuspended in DMEM supplemented with 10% FBS (Sigma–Aldrich) and 1% penicillin/streptomycin (P/S) at 37 °C with 5% CO_2_. The next day, BAT‐SVF cells were immortalized with the SV40 large T antigen according to a published protocol.^[^ ^32^ ^]^ For iBAT‐SVF cell differentiation, confluent SVF cells were induced to differentiate with medium containing 0.5 mm isobutylmethylxanthine (Sigma, Cat# I‐5879), 125 µm indomethacin (Sigma, Cat# I‐7378), 2 µg mL^−1^ dexamethasone (Sigma, Cat# D‐1756), 5 µg mL^−1^ insulin (Solarbio, Cat# I‐8040), 1 nm triiodo‐L‐thyronine (Sigma, Cat# T‐2877), and 1 µm rosiglitazone (Cayman, Cat# 71740‐100). After 48 h, the induction medium was replaced with a maintenance cocktail (5 µg mL^−1^ insulin, 1 nm triiodo‐L‐thyronine, and 1 µm rosiglitazone) until 6 to 8 days after the induction of differentiation.

### RNA Preparation, qPCR, and RNA‐Seq

Total RNA was extracted from tissue or cells via RNAiso Plus (Takara Biotech., Dalian, China, Cat# 9109) according to the manufacturer's instructions and quantified via a NanoDrop spectrophotometer. Reverse transcription of cDNA was completed via the use of 1 µg of RNA with HiScript II Q RT SuperMix for real‐time quantitative PCR (qPCR) (Vazyme Biotech, Nanjing, China, Cat# R222) according to the provided protocols. qPCR was performed with a real‐time PCR system on a QuantStudio1 (Applied Biosystems) using Taq Pro Universal SYBR qPCR Master Mix (Vazyme Biotech., Nanjing, China, Cat# Q712‐02). Relative transcript levels were calculated via the comparative *C*_T_ method; *36b4* was used as the internal control for cells, whereas *Hprt* was used as the internal control for tissues. The primer sequences are listed in Table S1 (Supporting Information). The mRNAs from the BAT of control and *Gabpα* ^Ucp1^ KO mice were extracted and subjected to commercial RNA‐seq (BGI Genomics Co., Ltd.) for further DEG and KEGG pathway enrichment analysis. In detail, RNA quality was examined with Fragment (Agilent). High‐quality RNA (RIN > 8.0) was used to library construction. RNA‐seq was performed at BGI Genomics (China) using the Illumina NovaSeq 6000 platform. Clean reads were aligned to the mouse reference genome (GRCm39) using Bowtie2, and transcript quantification was performed with RSEM. DESeq2 was used to determine differential genes, genes with an adjusted *p*‐value (FDR) < 0.05 and |log_2_FC| > 1 were considered significantly differentially expressed. The RNA‐seq data generated in this study have been deposited in the National Center for Biotechnology Information Sequence Read Archive database under accession code PRJNA1358056.

### Immunoblotting

Cells and tissues were lysed in RIPA lysis buffer (Beyotime, Shanghai, China, Cat# P0013B) containing a protease inhibitor cocktail (MedChem Express, NJ, USA, Cat# HY‐K0010) according to the manufacturer's protocol. Equal amounts of protein were separated on SDS‒PAGE gels and transferred to PVDF membranes (Millipore). The PVDF membranes were blocked in 5% skim milk for 1 h at room temperature and then incubated with primary antibody overnight at 4 °C. The membranes were washed three times with TBST and then incubated with HRP‐conjugated secondary antibodies for 1 h at room temperature. After washing three times, the Western blots were imaged via an enhanced chemiluminescence system.

The following antibodies were used for Western blotting: rabbit anti‐β‐actin (ABclonal, Wuhan, China, Cat# AC038, 1:10 000 dilution); rabbit anti‐Gabpα (Proteintech, Wuhan, China, Cat# 21542‐1‐AP, 1:1000 dilution); rabbit anti‐Pparγ (CST, USA, Cat# 2443, 1:1000 dilution); rabbit anti‐Eno1 (CST, USA, Cat# 3810, 1:1000 dilution); rabbit anti‐Ucp1 (Abcam, UK, Cat# ab234430, 1:1000 dilution); rabbit anti‐Ldha (ABclonal, Wuhan, China, Cat# A1146, 1:1000 dilution); rabbit anti‐Ldhb (ABclonal, Wuhan, China, Cat# A7625, 1:1000 dilution); rabbit anti‐Flag (Proteintech, Wuhan, China, Cat# 20543‐1‐AP, 1:1000 dilution); rabbit anti‐Myc (Proteintech, Wuhan, China, Cat# 16286‐1‐AP, 1:2000 dilution); rabbit anti‐His (Proteintech, Wuhan, China, Cat# 10001‐0‐AP, 1:1000 dilution); rabbit anti‐GST (Proteintech, Wuhan, China, Cat# 10000‐0‐AP, 1:1000 dilution); and HRP goat anti‐rabbit‐IgG (H+L) (ABclonal, Wuhan, China, Cat# AS014, 1:5000 dilution).

### Co‐Immunoprecipitation (Co‐IP)

To investigate the interaction between Gabpα and Pparγ, His‐Gabpα or the C‐terminal truncation mutants and Myc‐Pparγ plasmids were cotransfected into HEK293T cells, and cell extracts were generated after 72 h in lysis buffer (50 mm Tris‐HCl pH 7.4, 150 mm NaCl, 1% Triton X‐100, 1 mm EDTA, and protease inhibitor cocktail). Protein A/G magnetic beads (MedChem Express, NJ, USA, #HY‐K0202) were incubated with control IgG (2 µg, Proteintech, Wuhan, China, Cat# B900620) and primary antibodies against His (2 µg, Proteintech, Wuhan, China, Cat# 66005‐1‐Ig) with end‐end rotation at 4 °C for 2 h. Then, the cell supernatants were retained and incubated with the antibody‐magnetic beads complex with end‐end rotation overnight at 4 °C. For the other Co‐IP experiments, the cells were cotransfected with His‐Gabpα and the indicated plasmids. For the peptide GPCBi experiments, an additional peptide, GPCBi (30 µm), was added to the HEK293T cells for 48 h. For differentiated iBAT‐SVF cells, a Flag‐Gabpα lentivirus was transfected into SVF cells, which were subsequently differentiated into brown adipocytes. Protein A/G magnetic beads were incubated with control IgG and an anti‐Flag antibody (2 µg, Proteintech, Wuhan, China, Cat# 66008‐4‐Ig) and then incubated with the cell supernatants as described above. After four washes, the bound proteins were eluted, separated by SDS–PAGE, and analysed by immunoblotting. For the preparation of LC/MS‐MS, Flag‐GABPα plasmid was transfected into HEK293T cells for 72 h. The protein‐IgG/Flag antibody‐magnetic beads complex was subjected to LC/MS‐MS analysis by Bioprofile Biotechnology Co., Ltd (Shanghai, China). The protein mass spectrometry raw data has been deposited in ProteomeXchange partner repository under accession code PXD070466.

### Glutathione S‐Transferase (GST) Pulldown Assay

The purified proteins were prepared by Cusabio Technology, Inc. (Wuhan, China). The GST‐Gabpα fusion protein (1 µg) was incubated with glutathione magnetic agarose beads (MedChem Express, NJ, USA, #HY‐K0234) at 4 °C for 2 h and then incubated with equal amounts of translated His‐Pparγ protein (1 µg) at 4 °C overnight with end‐end rotation. After four washes, the eluted proteins were separated via SDS–PAGE and analysed by immunoblotting.

### Lipid Staining with Oil Red O

Differentiated iBAT‐SVF cells were washed twice with PBS, fixed in 4% paraformaldehyde for 30 min, and washed twice with PBS. The diluted Oil Red O solution (Oil Red O:ddH_2_O = 3:2, Oricell, Guangzhou, China, Cat# OILR‐10001) was allowed to set for 10 min and filtered through a 0.22 µm syringe filter. The cells were stained with oil red O solution for 30 min at room temperature and then washed three times with PBS, followed by imaging with a light microscope.

### Immunofluorescence

Differentiated iBAT‐SVF cells were fixed with 4% paraformaldehyde for 20 min at room temperature and then rinsed three times with PBS. The cells were permeabilized in 0.2% Triton X‐100 in PBS for 5 min and then washed three times with PBS. The cells were blocked with 3% BSA in PBS for 60 min and then incubated overnight at 4 °C with the primary antibodies rabbit anti‐Gabpα (Proteintech, Wuhan, China, Cat# 21542‐1‐AP, 1:100 dilution) and mouse anti‐Pparγ (Proteintech, Wuhan, China, Cat# 66936‐1‐Ig, 1:100 dilution). The cells were subsequently washed three times with PBS and incubated with a fluorescence‐conjugated secondary antibody (ABclonal, Wuhan, China, Cat# AS053; AS008, 1:200 dilution) for 1 h at room temperature. After washing three times, DAPI (BioLegend, USA, Cat# 422801) was used for nuclear counterstaining, and images were acquired via a Leica DMi8 confocal microscope at the Experimental Medicine Center of Tongji Hospital, Tongji Medical School, Huazhong University of Science and Technology.

### Luciferase Reporter Assay

HEK293T cells were transfected with 100 ng of Pparγ and 100 ng of Rxrα or empty vector together with 100 ng of Gabpα plasmid or the C‐terminal truncation mutants and 200 ng of *Eno1* luciferase reporter plasmid in 24‐well plates via Lipofectamine reagent (Yeasen, Shanghai, China, Cat# 40802ES03). For the experiments with the peptides GPCBi or T0070907, the additional peptides GPCBi (3 µm) or T0070907 (10 µm) were added to the HEK293T cells one day after the plasmid complex was transfected. After 48 h of transfection, luciferase activity was measured via the Dual‐Luciferase Reporter Assay System (Vazyme Biotech., Nanjing, China; Cat# DL101‐01). Firefly luciferase reporter gene measurements were normalized to Renilla luciferase activity.

### Tissue Histology and Immunohistochemistry

For haematoxylin and eosin (H&E) staining, adipose tissues and the liver were fixed in 4% paraformaldehyde overnight at 4 °C and then dehydrated in 70% ethanol. The tissues were subsequently embedded in paraffin, sectioned at a thickness of 5 µm, and stained with H&E or immunostained with a Ucp1 antibody (Abcam, UK, Cat# ab234430, 1:500 dilution) following the standard protocol. For Oil Red O staining, frozen liver sections were stained with Oil Red O solution for 10 min and then counterstained with haematoxylin for 20 s. Images of the tissue samples were captured via an Olympus BX53 Orthomicroscope. The color development time was optimized by monitoring the signal under the microscope.

### Oxygen Consumption and Glucose Stress Assays

The OCR and ECAR were measured in differentiated iBAT‐SVF cells via a Seahorse XFe Extracellular Flux Analyser (Agilent). For the mitochondrial stress test, differentiated iBAT‐SVF cells (control and sh*Gabpα*) were preincubated with XF assay medium at a pH of 7.4 for 1–2 h at 37 °C without CO_2_. The XF assay medium was supplemented with 1 mm sodium pyruvate, 2 mm glutamine, and 10 mm glucose. During each run, the cells were treated sequentially with oligomycin (4 µm), phenylhydrazone (FCCP, 4 µm), and rotenone and antimycin A (R&A, 1.35 µm). For the glycolysis stress test, XF assay medium was supplemented with only glutamine, and the cells were treated sequentially with highly concentrated glucose (10 mm), oligomycin (4 µm), and 2‐DG (50 mm). Data were analyzed using Seahorse Wave Desktop Software (Agilent).

### Metabolic Chamber Measurements

Three‐month‐old mice (*Gabpα* ^Ucp1^ KO, *Gabpα* ^Adipo^ KI, peptide GPCBi, and respective littermate control mice) were subjected to whole‐body energy expenditure measurements in metabolic chambers. Oxygen consumption and heat production were monitored via a Comprehensive Laboratory Animal Monitoring System (CLAMS, Columbus Instruments) at specific temperatures. *Gabpα* ^Ucp1^ KO and peptide GPCBi group mice were evaluated at ambient temperature, whereas *Gabpα* ^Adipo^ KI and their littermate control mice were evaluated with a temperature gradient from 30 to 23 °C and further to 4 °C. The VO2 and HEAT data were normalized to per mouse using ANCOVA analysis.^[^ ^55^ ^]^

### ^18^F‐FDG PET–CT

*Gabpα* ^Adipo^ KI and littermate control mice were exposed to a temperature of 30 °C for 7 days, followed by a temperature of 10 °C for 1 day, whereas *Gabpα* ^Ucp1^ KO and control mice were acclimatized at room temperature. The mice were administered ^18^F‐FDG via an intraperitoneal injection under 2% isoflurane anaesthesia after 6 h of fasting. PET‐CT scans were then performed via the integrated imaging system at the Laboratory of Nuclear Medicine.

### Lactic Acid Level Detection

Brown fat was homogenized with lysis buffer, ultrasonographically treated on ice at 300 W (on for 3 s, off for 7 s) for 2 min, and then centrifuged at 4 °C at 12 000 × g for 10 min. Serum and BAT lactate levels were measured with a lactic acid assay kit (Nanjing Jiancheng Bioengineering Institute, China, A019‐2‐1). All protocols were followed in accordance with the manufacturer's guidelines.

### Chromatin Immunoprecipitation and qPCR (ChIP‐qPCR)

ChIP assay was performed by using a ChIP assay kit (CST, USA, Cat# 9003). Detailed procedures were performed according to the manufacturer's instructions. The Flag antibody used for immunoprecipitation in this assay was obtained from Proteintech (Proteintech, Wuhan, China, Cat# 20543‐1‐AP). The qPCR assay was performed by using the primer targeting mouse *Eno1* promoter region, and the primer sequences are listed in Table S1 (Supporting Information).

### Surface Plasmon Resonance (SPR) Analysis

SPR analysis was performed using a Biacore T200 system (Cytiva) equipped with a Series S Sensor Chip CM5. Briefly, the ligand Pparγ protein was immobilized to the CM5 Chip via covalent bonds to the amino acid residues in immobilization buffer. Subsequently, different concentrations of Gabpα were diluted in the analyte buffer and were injected into the flowing channel to allow interaction between Pparγ and Gabpα. For a competitive binding assay, an equal concentration of Gabpα and different concentrations of GPCBi were injected into the flowing channel to examine the effect of the interaction between Pparγ and Gabpα. The interacting phase included 120 s of the association phase and 200 s of the dissociation phase. The data were analyzed in a Biacore T200 Evaluation Software (Cytiva).

### Statistical Analysis

All the data are expressed as the means ± s.e.m. and were analysed with the statistical software Prism v.9.0 (GraphPad Software, Inc., USA) and Microsoft Excel. RNA‐seq was performed once, but three independent samples were analysed and further validated via qPCR. Two‐tailed unpaired Student's *t* tests were used for two‐group comparisons. One‐way ANOVA followed by Tukey's test was used for comparisons of multiple groups. *p* < 0.05 was considered to indicate statistical significance throughout the study. No statistical methods were used to predetermine the sample size. For nonquantitative data, such as micrographs, the results were reproduced at least twice by replicating the experiments, and representative results are shown.

## Conflict of Interest

The authors declare no conflict of interest.

## Author Contributions

Z.W., H.W., Q.K., and R.P. contributed equally to this work. Y.C. conceived the study. Z.W., H.W., Q.K., R.P., R.H., M.Y., J.L., X.P., Y.X., H.D., W.W., Z.Z., J.G., Y.L., and R.G. designed and performed experiments and analyzed the data. Y.G., P.Y., L.P., and D.L. provided materials for the experiments in vitro. Z.W., H.W., Q.K., R.P., and Y.C. wrote the manuscript. P.M., X.L., and X.Y. provided suggestions for the revision. Y.C., R.P., Q.K., and P.M. edited the manuscript. All authors read and approved the revised manuscript. Y.C. supervised and led this work.

## Supporting information



Supporting Information

Supporting Information

## Data Availability

The data that support the findings of this study are available from the corresponding author upon reasonable request.
