# Supplementary material for: Gabpα‐Pparγ Complex Determines Glycolytic Capacity and Lactic Acid Homeostasis in Brown Fat
Source: Adv Sci (Weinh). 2025 Nov 23;13(8):e17426. doi: 10.1002/advs.202517426 (PMC12884720; doi:10.1002/advs.202517426)
Supplement: Supplementary file 1 — Supporting Information [file ADVS-13-e17426-s002.pdf]

Supporting Information

Gabpα-Pparγ Complex Determines Glycolytic Capacity and Lactic Acid Homeostasis in Brown Fat

Zhihan Wang, Huanyu Wang, Qianqian Kang, Ruping Pan, Rui He, Min Yang, Jiadai Liu, Xuemin Peng, Yuyu Xie, Hongyan Deng, Wenshe Wang, Zengzhe Zhu, Jing Ge, Yulian Liu, Ronghui Gao, Yaming Guo, Peng Yu, Limeng Pan, Danpei Li, Pema Maretich, Xiaoping Luo, Xuefeng Yu, and Yong Chen[*]

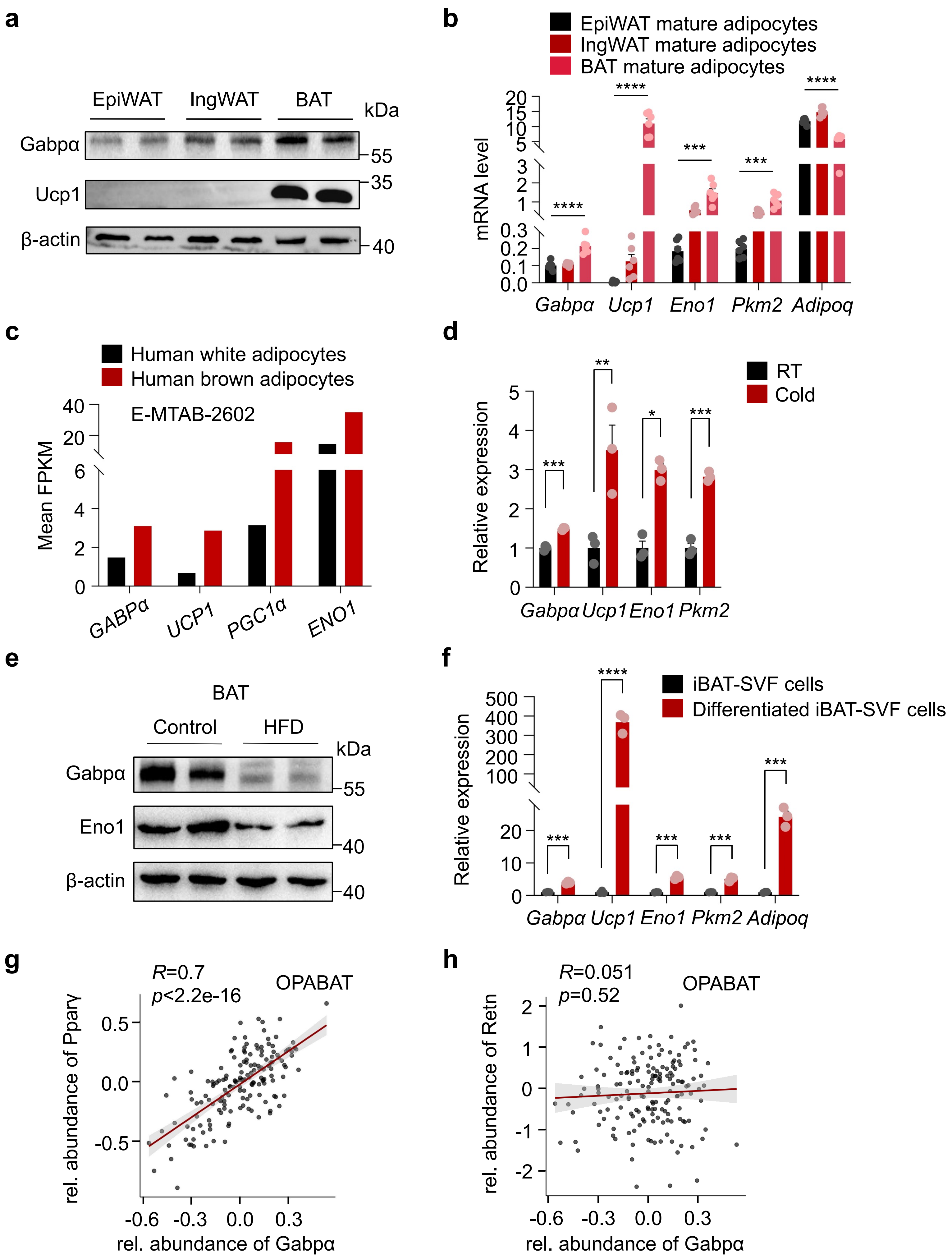

Figure S1. Gabpα is related to BAT glycolytic capacity (related to Figure 1).

a) Western blot analysis of Gabpα and Ucp1 in BAT, IngWAT, and EpiWAT from wild-type mice. b) Relative mRNA levels of related genes in primary mature adipocytes isolated from BAT, IngWAT, and EpiWAT from three-month-old male mice (n = 6). c) Mean FPKM (Fragments Per Kilobase of transcript per Million mapped reads) values of related genes in human brown and white adipocytes according to the Supplementary Table of published data (E-MTAB-2602). d) Relative mRNA levels of related genes in BAT from three-month-old male mice maintained at room temperature and exposed to cold temperatures (4 °C) for 48 h (n = 3). e) Western blot analysis of Gabpα and Eno1 in BAT from normal diet-fed and twelve-week HFD-fed mice. f) Relative mRNA levels of related genes in iBAT-SVF cells and differentiated iBAT-SVF cells (n = 3). g,h) Relative abundances of Gabpα and Pparγ (g) or Retn (h) in 163 fully genotyped diverse outbred mice from the OPABAT website. The data are presented as the means ± s.e.m. *$p < 0.05$, **$p < 0.01$, ***$p < 0.001$, and ****$p < 0.0001$ according to a two-tailed unpaired Student's *t* test.

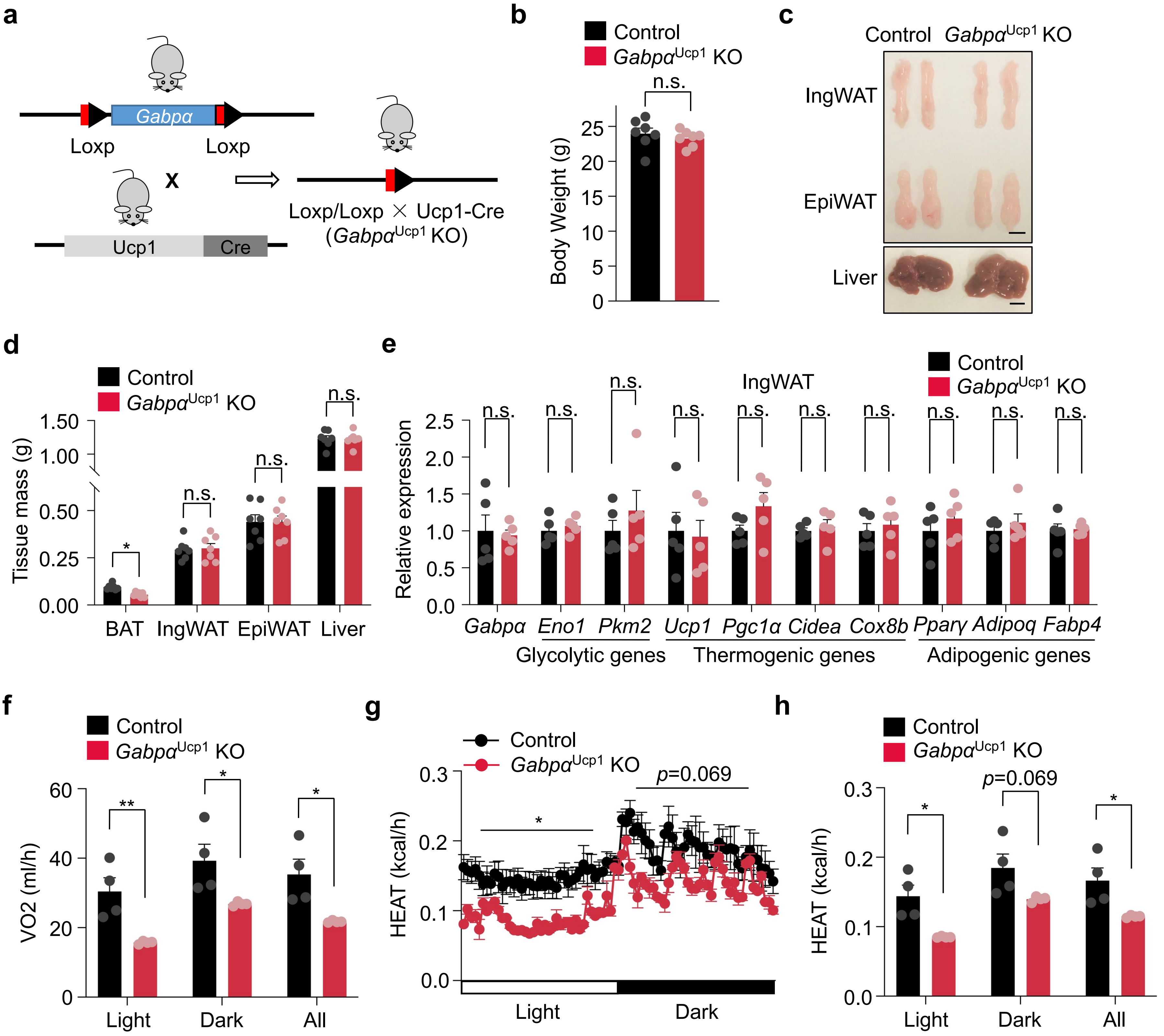

Figure S2. Deletion of Gabpα in BAT impairs glycolysis and energy expenditure (related to Figure 2).

a) Overview of the *Gabpα*^{Ucp1} KO mouse model. b) Body weights of control and *Gabpα*^{Ucp1} KO mice (three months old) (n = 7). c) Morphology of the fat pads and liver of control and *Gabpα*^{Ucp1} KO mice fed a chow diet. Scale bar, 5 mm. d) Tissue masses of BAT, IngWAT, EpiWAT, and liver from control and *Gabpα*^{Ucp1} KO mice (three months old) (n = 7). e) Relative mRNA levels of related genes in IngWAT from control and *Gabpα*^{Ucp1} KO mice (n = 5). f-h) Oxygen consumption (f) and heat production (g,h) of control and *Gabpα*^{Ucp1} KO mice housed at 28 °C (n = 4). The data are presented as the mean ± s.e.m. *p < 0.05 and **p < 0.01 according to a two-tailed unpaired Student's t test.

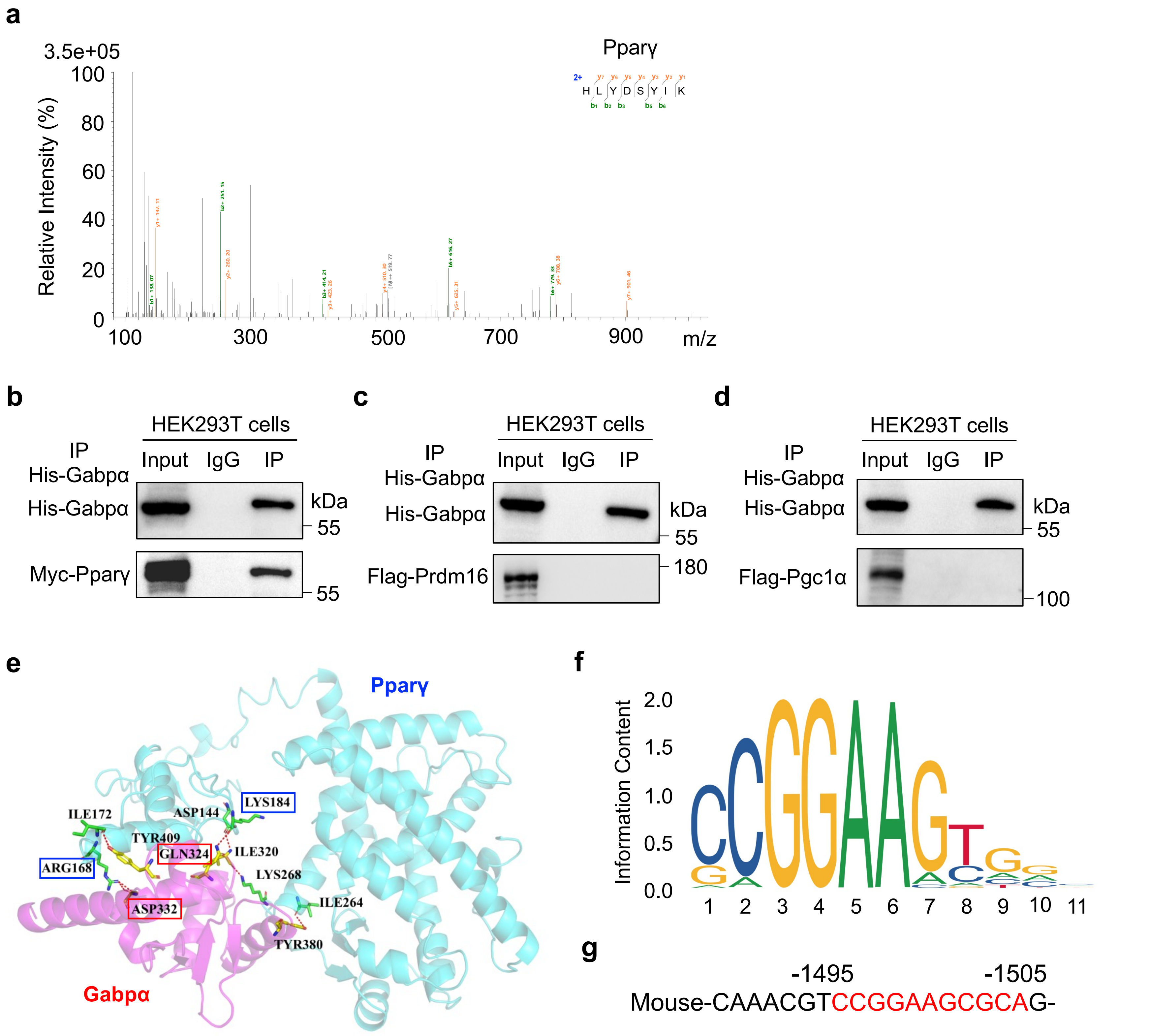

Figure S3. Gabpα binds to Pparγ to increase the transcription of the glycolytic gene *Eno1* (related to Figure 3).

a) Identification of Pparγ as an interactor of Gabpα by LC-MS/MS. b-d) Co-IP was used to investigate the physical interactions between Gabpα and the Pparγ (b), Prdm16 (c), and Pgc1α (d) proteins in cell lysates from HEK293T cells transfected with the indicated plasmids. IgG, immunoglobulin G; IP, immunoprecipitation. e) Visualization of protein structures and protein docking site prediction of Gabpα and Pparγ via the HDOCK server. f) Position weight matrix of the canonical Gabpα DNA binding motif from the JASPAR database. g) A canonical Gabpα binding site sequence identified in the mouse *Eno1* gene.

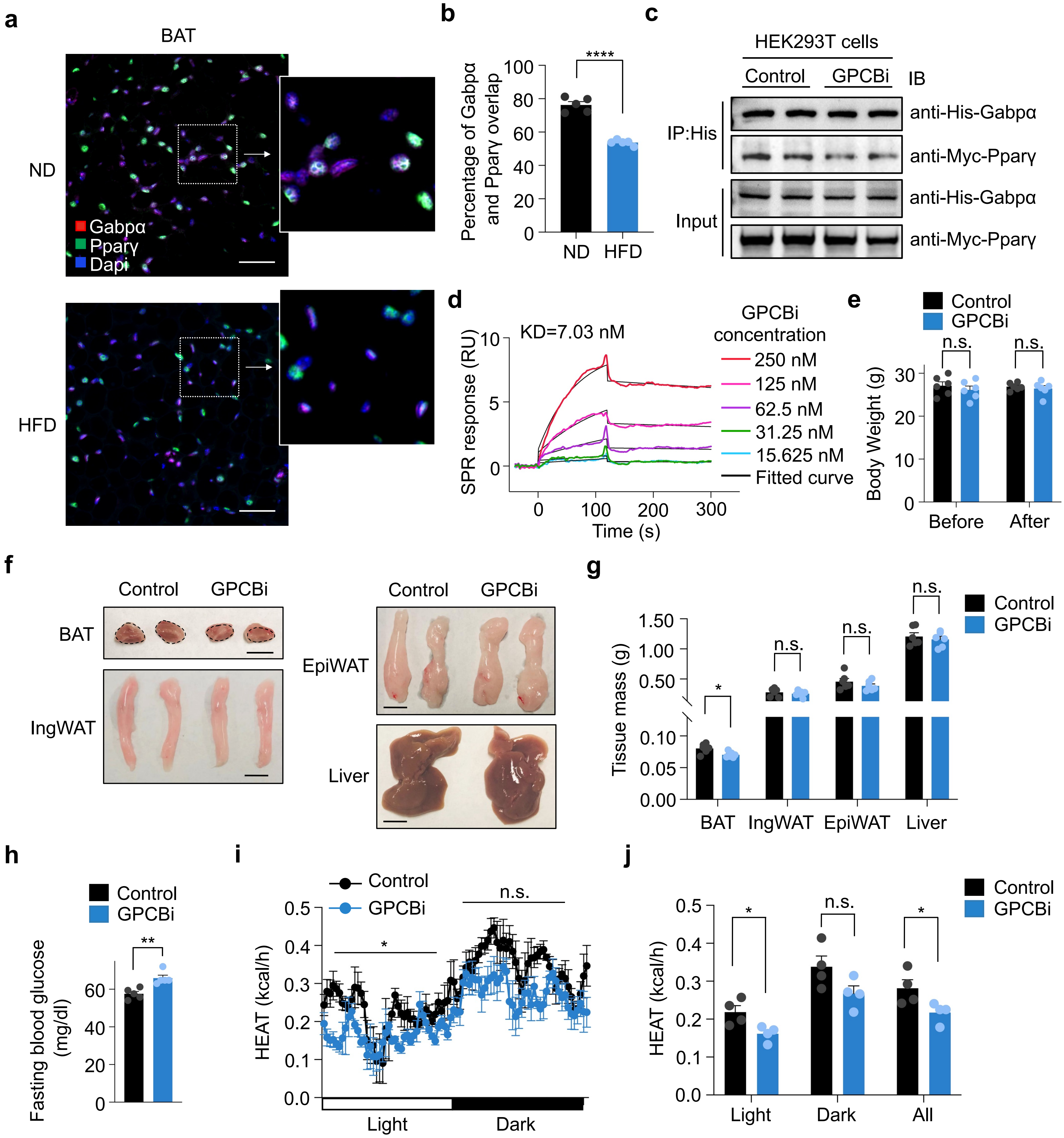

Figure S4. Inhibition of Gabpα-Pparγ binding represses BAT capacity (related to Figure 4).

a) Immunofluorescence analysis of the colocalization of Gabpα and Pparγ in the nuclei of BAT from normal diet-fed and twelve-week HFD-fed mice. Scale bar, 50 μm. b) Quantification of the percentage of Gabpα and Pparγ colocalization in Figure S4a analyzed by Image J. c) Investigation of the effects of the peptide GPCBi via CoIP assays in HEK293T cells transfected with the indicated plasmids. IP, immunoprecipitation. d) SPR sensorgrams showing the interaction between different concentrations of GPCBi and Gabpα protein. e) Body weights of wild-type mice before and after the peptide GPCBi intervention (n = 4). f) Morphology of BAT, IngWAT, EpiWAT, and liver from wild-type mice treated with the peptide GPCBi. Scale bar, 5 mm. g) Tissue masses of BAT, IngWAT, and EpiWAT and liver of wild-type mice treated with the peptide GPCBi (n = 4). h) Fasting blood glucose of wild-type mice treated with the peptide GPCBi (n = 5). i,j) Heat production of wild-type mice treated with the peptide GPCBi (n = 4). The data are presented as the means ± s.e.m. *p < 0.05, **p < 0.01, and ****p < 0.0001 according to a two-tailed unpaired Student's t test.

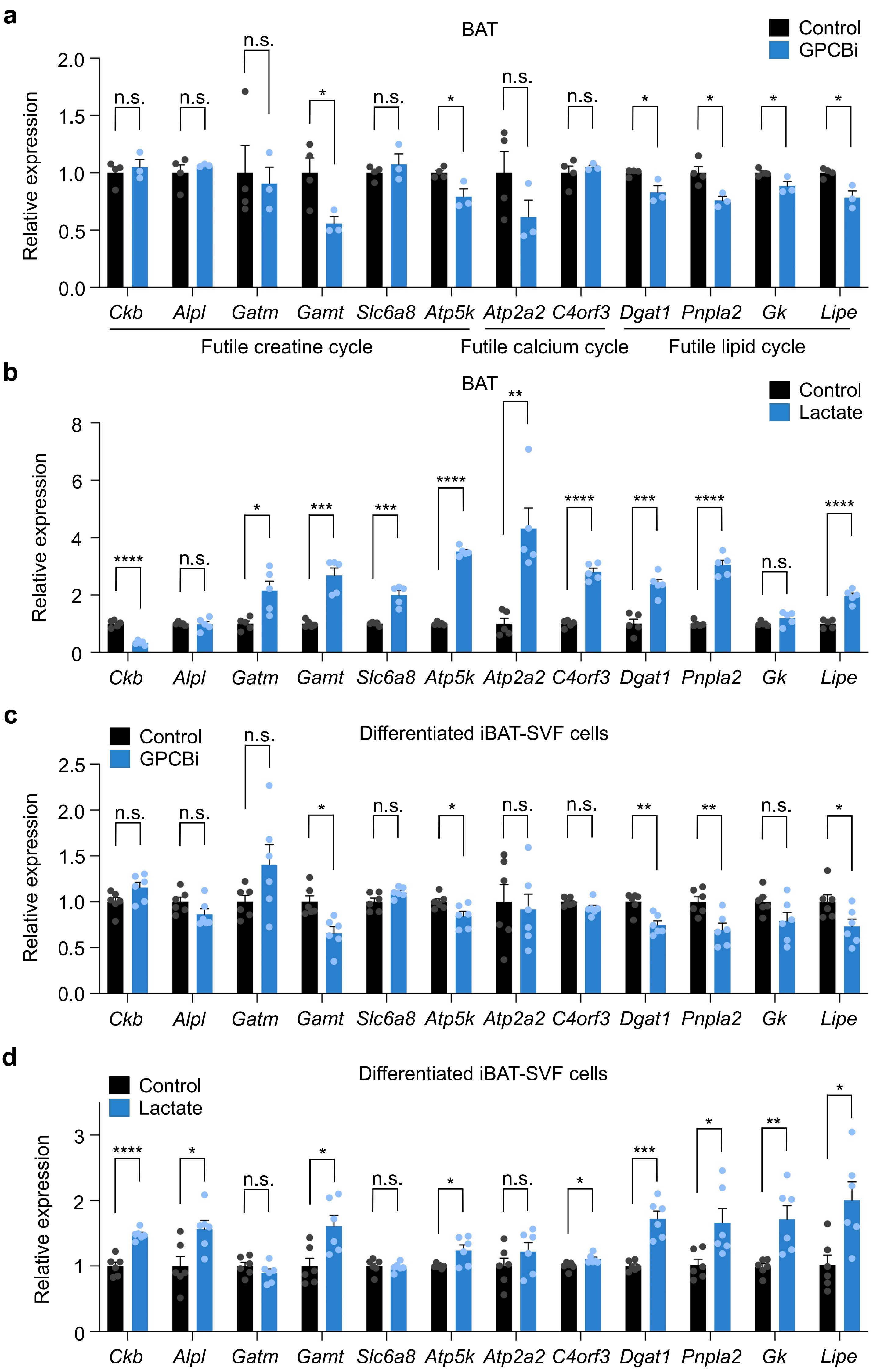

Figure S5. The regulatory gene expression of the futile cycles in the interventions of GPCBi and lactate (related to Figure 5).

a) Relative mRNA levels of futile cycles in BAT from wild-type mice treated with the peptide GPCBi at 23 °C for 7 days to 10 °C for 2 days (control, n = 4; GPCBi, n = 3). b) Relative mRNA levels of futile cycles in BAT from wild-type mice treated with intraperitoneally injected sodium lactate (2 g kg^{-1} d^{-1}) daily for 3 days. c) Relative mRNA levels of futile cycles in differentiated iBAT-SVF cells after intervention with the peptide GPCBi (5 μM) during the first 2 days of adipogenic differentiation. d) Relative mRNA levels of futile cycles in differentiated iBAT-SVF cells after intervention with sodium lactate (30 mM) for 24 h. The data are presented as the means ± s.e.m. *p < 0.05, **p < 0.01, ***p < 0.001, and ****p < 0.0001 according to a two-tailed unpaired Student's t test.

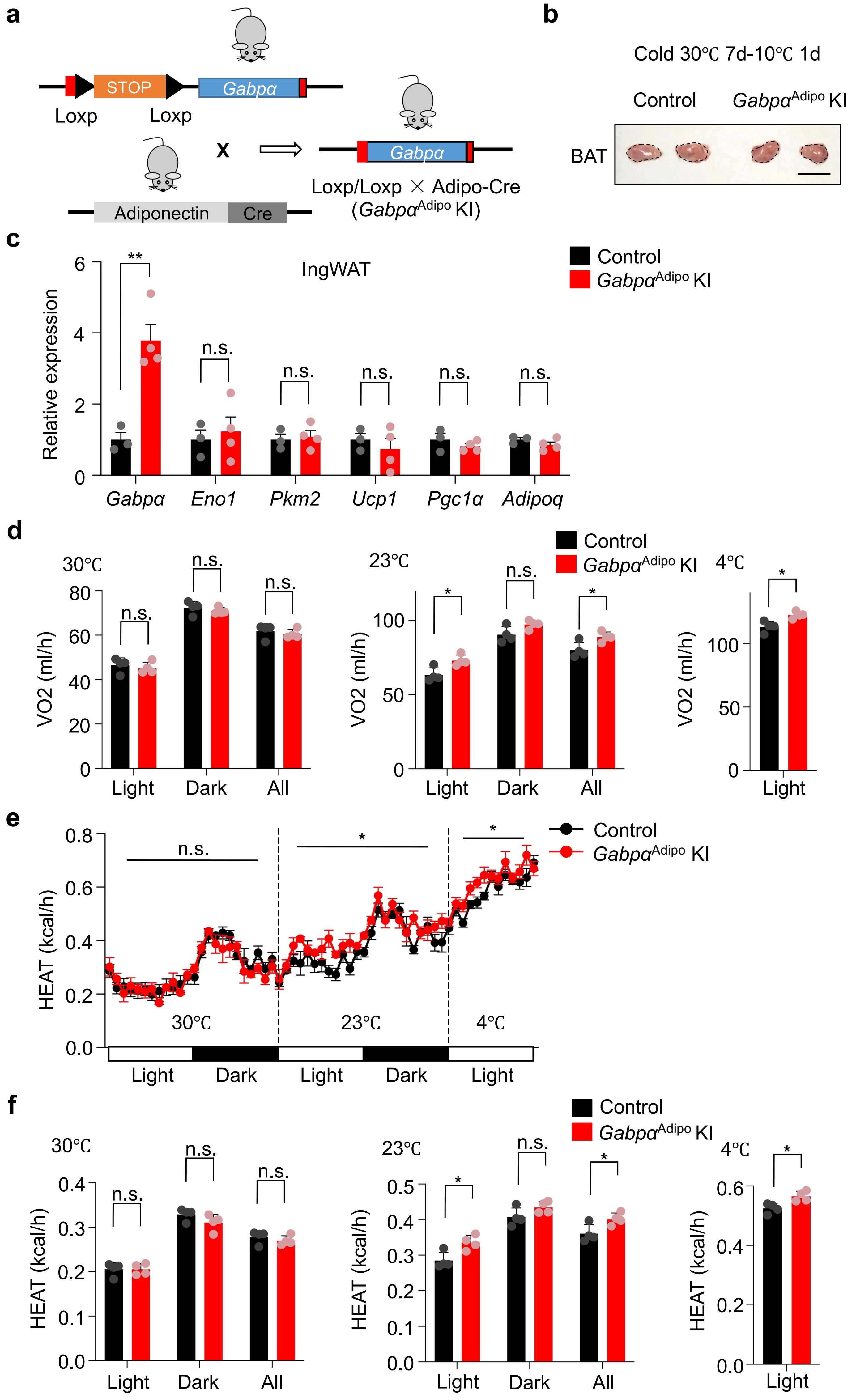

Figure S6. Adipocyte-specific Gabpα overexpression enhances glycolytic capacity in BAT (related to Figure 6).

a) Overview of the *Gabpα*Adipo KI mouse model. b) Morphology of BAT from control and *Gabpα*Adipo KI mice after switching from 30 °C for 7 days to 10 °C for 1 day. Scale bar, 5 mm. c) Relative mRNA levels in IngWAT from control and *Gabpα*Adipo KI mice after a switch from 30 °C for 7 days to 10 °C for 1 day (control, n = 3; *Gabpα*Adipo KI, n = 4). d-f) Oxygen consumption (d) and heat production (e,f) of control and *Gabpα*Adipo KI mice housed at a temperature gradient from 30 °C to 23 °C and further to 4 °C (n = 4). The data are presented as the mean ± s.e.m. *p < 0.05 and **p < 0.01 according to a two-tailed unpaired Student's t test.

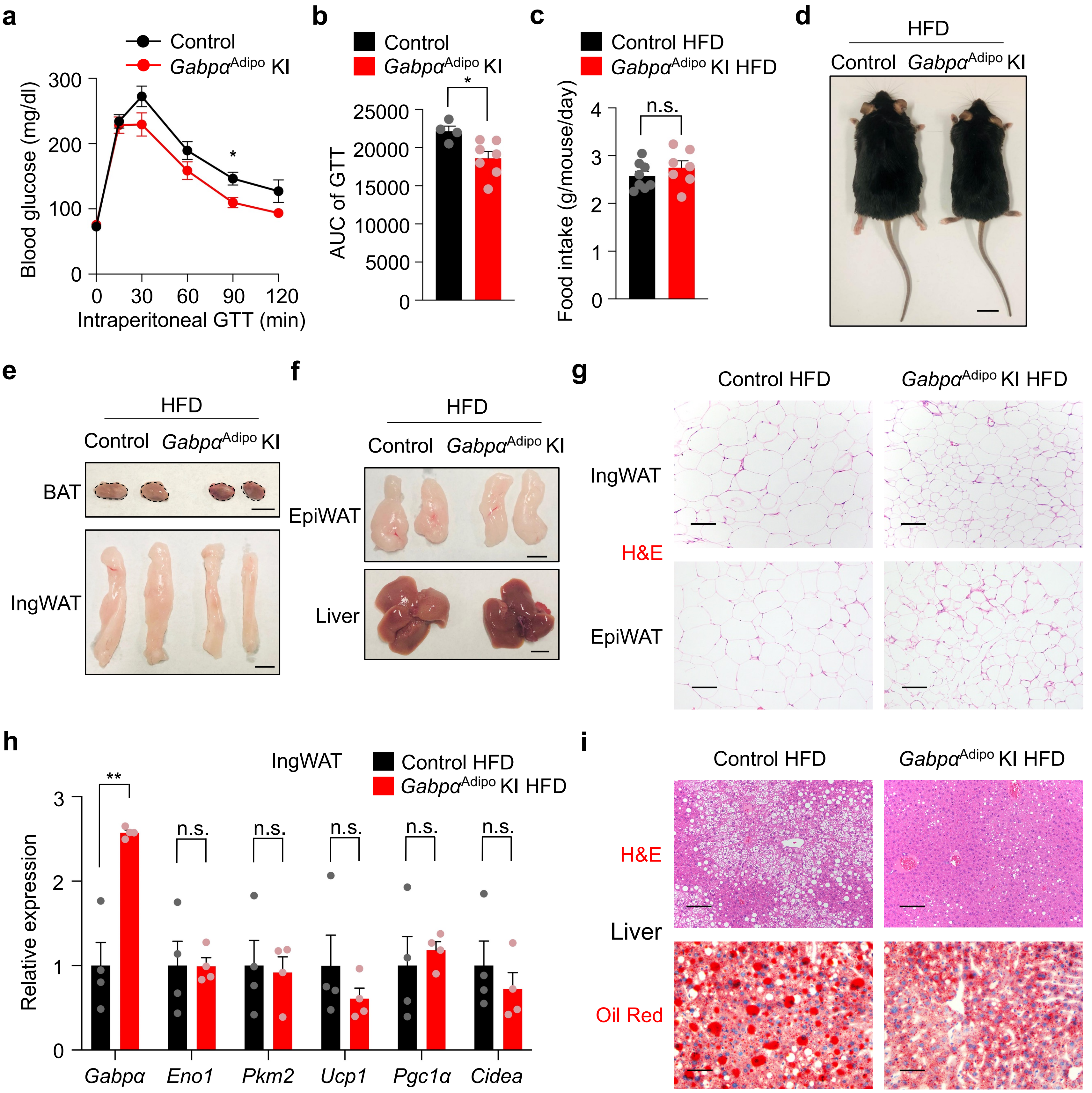

Figure S7. Adipocyte-specific Gabpα overexpression protects against HFD-induced obesity (related to Figure 6).

a) Glucose tolerance test of control and *Gabpα*^{Adipo} KI mice fed a normal diet (control, *n* = 4; *Gabpα*^{Adipo} KI, *n* = 7). b) Area under the curve (AUC) quantification of the glucose tolerance test results in Figure S7a. c) Food intake of control and *Gabpα*^{Adipo} KI mice fed a twelve-week HFD (control, *n* = 8; *Gabpα*^{Adipo} KI, *n* = 7). d-f) Representative photographs of control and *Gabpα*^{Adipo} KI mice (d), fat pads and liver (e,f) after a twelve-week HFD. d, Scale bar, 1 cm; e, f, Scale bar, 5 mm. g) H&E staining of IngWAT and EpiWAT from control and *Gabpα*^{Adipo} KI mice after a twelve-week HFD. Scale bar, 100 μm. h) Relative mRNA levels of related genes in IngWAT from control and *Gabpα*^{Adipo} KI mice after a twelve-week HFD (*n* = 4). i) H&E staining and Oil Red O staining of the liver of control and *Gabpα*^{Adipo} KI mice after a twelve-week HFD. Scale bar, H&E staining, 100 μm; Oil Red O staining, 50 μm. The data are presented as the means ± s.e.m. $^*p < 0.05$ and $^{**}p < 0.01$ according to a two-tailed unpaired Student's *t* test.
