# Supplementary material for: Gabpα‐Pparγ Complex Determines Glycolytic Capacity and Lactic Acid Homeostasis in Brown Fat
Source: Adv Sci (Weinh). 2025 Nov 23;13(8):e17426. doi: 10.1002/advs.202517426 (PMC12884720; doi:10.1002/advs.202517426)
Supplement: Supplementary file 2 — Supporting Information [file ADVS-13-e17426-s001.docx]

**Table S1. Sequences of primers used for real-time quantitative PCR (qPCR)**

| Genes | Forward primer | Reverse primer |
| --- | --- | --- |
| *Gabpα* | AGCGCATCTCGTTGAAGAAG | TCCTGCTCTTTTCTGTAGCCT |
| *Ucp1* | CACCTTCCCGCTGGACACT | CCCTAGGACACCTTTATACCTAATGG |
| *Pgc1α* | AGCCGTGACCACTGACAACGAG | GCTGCATGGTTCTGAGTGCTAAG |
| *Cox8b* | GAACCATGAAGCCAACGACT | GCGAAGTTCACAGTGGTTCC |
| *Eno1* | TGCGTCCACTGGCATCTAC | CAGAGCAGGCGCAATAGTTTTA |
| *Pkm2* | CGCCTGGACATTGACTCTG | GAAATTCAGCCGAGCCACATT |
| *Adipoq* | GGTCTTCTTGGTCCTAAGGGTG | TGCCGTCATAATGATTCTGTTG |
| *Fabp4* | AAGGTGAAGAGCATCATAACCCT | TCACGCCTTTCATAACACATTCC |
| *Retn* | ACAAGACTTCAACTCCCTGTTTC | TTTCTTCACGAATGTCCCACG |
| *Pparγ* | GGAAGACCACTCGCATTCCTT | GTAATCAGCAACCATTGGGTCA |
| *Ldha* | TATCTTAATGAAGGACTTGGCGGATGAG | GGAGTTCGCAGTTACACAGTAGTC |
| *Ldhb* | TTGTGGCCGATAAAGATTACTCTGTGAC | AGGAATGATGAACTTGAACACGTTGAC |
| *Cidea* | TGCTCTTCTGTATCGCCCAGT | GCCGTGTTAAGGAATCTGCTG |
| *Ckb* | GCCTCACTCAGATCGAAACTC | GGCATGTGAGGATGTAGCCC |
| *Alpl* | TCAACACCAATGTAGCCAAGA | GTAGCTGGCCCTTAAGGATTC |
| *Gatm* | ATGCCTGTGTGCCACCATTC | TTGCACATCTCTTCGACCTCA |
| *Gamt* | GCAGCCACATAAGGTTGTTCC | CTCTTCAGACAGCGGGTACG |
| *Slc6a8* | TGCATATCTCCAAGGTGGCAG | CTACAAACTGGCTGTCCAGA |
| *Atp5k* | GGTCACGGACAAAATGGTGC | GTCATCTTGAGCTTCCGCCA |
| *Atp2a2* | GCTCATTTTCCAGATCACACCG | GTTACTCCAGTATTGCGGGTTG |
| *C4orf3* | GCTGGATCTCTGGCTCTTCA | TCGTTTTCAAGATTCACTCCAG |
| *Dgat1* | GGAATATCCCCGTGCACAA | CATTTGCTGCTGCCATGTC |
| *Pnpla2* | GGAGGAATGGCCTACTGAACC | ATCCTCTTCCTGGGGGACAA |
| *Gk* | TCGTTCCAGCATTTTCAGGGTTAT | TCAGGCATGGAGGGTTTCACTACT |
| *Lipe* | AGGGAGGGCCTCAGCG | TTGGCTGGTGTCTCTGTGTC |
| *36b4* | TCCAGGCTTTGGGCATCA | CTTTATCAGCTGCACATCACTCAGA |
| *Hprt* | TCAGTCAACGGGGGACATAAA | GGGGCTGTACTGCTTAACCAG |
| *Eno1-Chip* | CTATGTAGACCAGGCAGGCC | TGATGTGACAAACGTCCGGA |
